# Repurposing the hypoglycaemic agents for neuroinflammation, a comprehensive review

**DOI:** 10.1007/s13205-025-04455-7

**Published:** 2025-08-05

**Authors:** Vandana Blossom, Sheetal D. Ullal, Rajalakshmi Rai, Melisha Michael D souza, P. Gopal Govind Kalluraya, Ayush Dixit, P. J. Jiji, B. V. Murlimanju

**Affiliations:** 1 Department of Anatomy, Kasturba Medical College Mangalore, Manipal Academy of Higher Education, Manipal, India; 2 Department of Pharmacology, Kasturba Medical College Mangalore, Manipal Academy of Higher Education, Manipal, India; 3 DrNB Neurosurgery, Medical Trust Hospital, Ernakulam, Kerala India; 4 Department of Hand Surgery, Kasturba Medical College, Manipal Academy of Higher Education, Manipal, India

**Keywords:** Diabetes mellitus, Hypoglycaemic agents, Neurodegenerative diseases, Neuroinflammatory diseases

## Abstract

The shared pathways between neuroinflammation and diabetes mellitus involve the NLRP3 inflammasome and subsequent production of the IL-1β. Chronic hyperactivation of hypothalamo-pituitary–adrenal axis and innate immunity are implicated in neurological disorders and diabetes. Repurposing drugs with anti-inflammatory properties allows for faster clinical translation in neuroinflammation as compared to developing new drugs from scratch. Few repurposed drugs have already undergone safety and efficacy testing for other conditions, making them attractive candidates for the neuroinflammatory disorders. Gliburide, an oral hypoglycaemic effectively inhibits the NLRP3 inflammasome, signifying that it may be used to treat the neuroinflammation-related disorders. A GLP-1 receptor agonist, liraglutide established encouraging effects in regulating hyperglycaemia and possibly lowering neuroinflammation. Patients who were obese and receiving liraglutide saw improvements in their glycaemic control and a decrease in neuroinflammatory markers in addition to the weight loss. Studies on mice suggested that, sulphonyl-ureas have properties to decrease the neuroinflammatory conditions and has potential benefits by targeting the NLRP3 inflammasome pathway, modulating lipopolysaccharide induced micro and astroglial neuroinflammation by activating the ERK/STAT3/NF-κB signalling pathways. Empagliflozin offered neuroprotection and helped in neurovascular remodelling, which is crucial for maintaining cognitive function. Repurposing is already-approved for the antidiabetic medications, such as insulin, metformin and thiazolidinediones. Insulin may be a viable and effective approach to treat neuroinflammation. In conclusion, the interplay between diabetes and neuroinflammation highlights the importance of metabolic health in neurodegenerative diseases. Understanding these shared pathways can inform strategies for prevention and treatment, potentially targeting both conditions simultaneously.

## Introduction

Whenever our body perceives potential insult or harm, it reacts naturally by releasing immune cells and causes inflammation in that site (Mukhara et al. 2020; Carson et al. 2006; Lo et al. 1999). The neuroinflammation occurs initially to overcome the deleterious effects of infection, ischemia, stress and trauma. The A2 astrocytes, M2 microglia and endothelial cells, which release the inflammatory cytokines and chemokines, take part in this process (Gorji 2022; Ní Chasaide and Lynch 2020). Neuroinflammation helps to maintain the brain homeostasis apart from fighting pathogens, repairing neurons and supporting their survival. Acute neuroinflammation could be beneficial or even protective, but chronic inflammation can be far from it. Multiple clinical and experimental studies have indicated that neuroinflammation can be a common link in various neurological pathologies like cerebrovascular accidents, multiple sclerosis, traumatic injuries of brain and spinal cord, neurodegeneration, behavioural disorders and epilepsy (Gorji 2022; Ashayeri Ahmadabad et al. 2020; Ní Chasaide and Lynch 2020; Gilhus and Deuschl 2019). Various elements that affect the way, an individual human responds to neuroinflammation, include the pathogens, environmental factors, genetic background and age (Carson et al. 2006). Intricate cellular and molecular pathways that contribute to the neuroinflammation also include infiltrated peripheral immune cells and finally interaction between the nervous and immune systems (Carson et al. 2006).

## Region-specific energy regulation in the brain in neurodegeneration

Alzheimer’s disease (AD) corners a considerable break on global healthcare systems, specifically affecting the geriatric population (Singh et al. 2024). It is characterized by impaired mitochondrial function, leading to energy deficits in neurons. The downregulation of mitochondrial NADH-dehydrogenase subunit genes, which are part of complex I of the electron transport chain (ETC), has significant implications for neurodegeneration, particularly in the cerebellum and AD. Different brain regions exhibit varying levels of oxidative stress and metabolic demand. The cerebellum's resilience highlights the importance of region-specific energy regulation in neurodegenerative diseases (Cenini and Voos 2019; Mattson and Magnus 2006; Swerdlow et al. 2014). Altered regulation of complex I genes could exacerbate this energy crisis in brain regions like the hippocampus and cerebral cortex, which are more energy-dependent than the cerebellum (Bubber et al. 2005; Swerdlow & Khan 2004) and lead to mitochondria dysfunction (Mattson and Magnus 2006). The lower ROS production mitigates oxidative damage, which is a critical driver of neurodegeneration.(Lin and Beal 2006; Yadav et al. 2022). Hence modulating the drugs targeting the mitochondrial biogenesis or alternative energy pathways like ketone metabolism, could help restore balance in the AD-affected regions. (Onyango et al. 2016). It was also opined that, no single drug was successful in controlling the symptoms of AD and management has been a difficult task (Tripathi et al. 2024). The dual-functional perspective of mitochondria enhances the understanding of AD progression by elucidating how mitochondrial dysfunction contributes to neuronal damage and how its maintaining can offer neuroprotection. This underscores the potential of targeting mitochondrial pathways as a therapeutic strategy for AD.

The literature search did not reveal evidence of shared genes with differential expression across the hippocampus, entorhinal cortex, cerebellum and cingulate gyrus in AD. But there is significant role of tau pathology and gene expression changes in the hippocampus and entorhinal cortex. Cingulate gyrus is also involved in the AD pathology, suggesting the complex interplay of gene expression and neurodegeneration across these regions. However, further research is needed to identify specific shared genes and their differential expression patterns in these brain regions. Cerebellum undergoes changes in AD, however its relative preservation as compared to other regions of brain may be due to intrinsic survival pathways, slower accumulation of genetic markers of aging, and specific epigenetic factors.

The pliability of cerebellum to the degenerative changes in diseases like AD was analysed with the rest of the regions of brain with the help of various experimental controls like quantitative measurements (Latimer et al. 2019), mapping (Gellersen et al. 2021), functional analysis (Tang et al. 2021) and sequencing of RNA (Hampel et al. 2021). Categorization of upregulated and downregulated genes is basic in understanding the neuroprotective profile of the cerebellum. This categorization assists in recognizing the genetic and molecular basis of cerebellum's resilience against the apoptosis, neuroinflammation, and neurodegeneration. For example, upregulation of heat shock proteins protects the neurons from apoptosis by managing the misfolded proteins (Qu et al. 2018). Another example is the downregulation of Wnt/β-catenin pathway by the drug, pioglitazone has shown to ameliorate the neuroinflammation and apoptosis, suggesting its neuroprotective role (Sandhu et al. 2024). Gene ontology analysis has discovered that, upregulated genes are instrumental in promoting the neurogenesis, cell survival, and antiapoptotic pathways. The downregulated genes will decrease the inflammation, cell death, and neurodegeneration. It was reported that, specific pathways like oxidative phosphorylation, FOXO and notch signalling are involved in the neuroprotection (Hu et al. 2015).

The micro-RNAs are crucial in upregulating and downregulating the expression of genes in neurodegeneration and their expression can be done by gene sequencing using the transgenic mouse model (Paul et al. 2024). The differential gene expression, integration of multiple datasets, comparative genomics, functional relevance, experimental validation, genetic and epigenetic factors were utilized by various scientists to select the appropriate genes for the neurorestoration (Zimmermann et al. 2017; Lazic 2015; Genter et al. 2003). The neuroprotective pathways in the different regions of brain including hippocampus, cingulate gyrus, cerebellum and entorhinal cortex could be differentiated by using the techniques like mass spectrometry and liquid chromatography. These will offer detailed analysis of the protein expression and post-translational modifications, which enable to understand the various mechanisms involved in the neuroprotection at various regions of brain (Xu et al. 2019). The gene expression analyses at multiple time points can offer a comprehensive understanding of the cerebellum's neuroprotective response in the progression of AD. The temporal changes and region-specific gene expression patterns could be studied to understand the molecular mechanisms and helps in identifying the novel therapeutic targets to alleviate the progression of AD (Wang et al. 2024). The assortment of differentially expressed 120 cerebellar genes in AD involves a combination of network and bioinformatics analyses, enrichment studies, and experimental validation to ensure their relevance to disease progression and potential as therapeutic targets (Feng et al. 2015).

The amyloid precursor-like protein 1 (APLP1), Thy-1 cell surface antigen (THY1), growth associated protein 43 (GAP 43), neural cell adhesion molecule 1 (NCAM1), synuclein beta (SNCB) and are the proteins, which play an important role in the neuroprotection and restoration by involving in the neuronal growth and synaptic plasticity. Their expression is obvious in the growth cones (Holahan et al. 2017; Flamm et al. 2016; Rosskothen-Kuhl et al. 2015; Rosskothen-Kuhl et al. 2014; Zhao et al. 2012; Kowara et al. 2007). GAP43 is important and widely expressed during the embryogenesis of the central nervous system, particularly in Cornu Ammonis region-3 and auditory regions and the postnatal development of cerebellum (Rosskothen-Kuhl et al. 2015; Rosskothen-Kuhl et al. 2014). Abnormal expression of NCAM1 can lead to excessive migration of Purkinje cells, which suggests the role of NCAM1 in the neuronal integrity (Shabanipour et al. 2022). The evolutionary theory of mitochondria provides valuable insights into their role in AD and highlights the potential for developing targeted therapeutic strategies. By focusing on mitochondrial repair, protection, and functional enhancement, researchers aim to mitigate the neurodegenerative processes in AD, offering hope for more effective treatments in the future.

### Diabetes mellitus and pathophysiology of hyperglycaemia

Development of hyperglycaemia can be attributed to a complex interplay of several key pathophysiological mechanisms, often referred to as the ‘ominous octet’: (Chaudhury et al. 2017; Defronzo 2009). These mechanisms, which may act alone or in combination, are as follows.

#### Insulin deficiency

The β cells present at the pancreas fail to produce sufficient insulin **(**Chaudhury et al. 2017; Defronzo 2009).

#### Elevated secretion of glucagon

The α-cells of pancreas produce excessive glucagon, which increases the blood glucose levels (Chaudhury et al. 2017; Defronzo 2009).

#### Increased hepatic glucose production

The liver generates an excessive amount of glucose (Chaudhury et al. 2017; Defronzo 2009).

#### Dysfunction of neuro-transmitters and brain insulin resistance

The brain's neurotransmitter activity is impaired, contributing to insulin resistance (Chaudhury et al. 2017; Defronzo 2009).

#### Enhanced lipolysis

There is an increased breakdown of fats, leading to elevated free fatty acids in the blood. (Chaudhury et al. 2017; Defronzo 2009).

#### Increased renal glucose reabsorption

The kidneys reabsorb more glucose back into the bloodstream (Chaudhury et al. 2017; Defronzo 2009).

#### Reduced incretin effect in the small intestine

The small intestine's incretin hormones, which normally enhance insulin secretion, are less effective (Chaudhury et al. 2017; Defronzo 2009).

#### Impaired peripheral tissue glucose uptake

Organs like liver, skeletal muscles and adipose tissue have a diminished capacity to absorb glucose (Chaudhury et al. 2017; Defronzo 2009).

### Diabetes and neuroinflammation

Elevated blood glucose due to insufficient insulin or insulin resistance causes a serious metabolic disease called diabetes mellitus. (Kim et al. 2016; Ramos-Rodriguez et al. 2014). It is a noncommunicable disease, where the patient suffers from hyperglycaemia either due to deficiency of insulin or inefficient action of insulin, which can lead to complications if left untreated or poorly treated (Chaudhury et al. 2017). Such type of chronic illness can alter the micro and macrovascular functions in the nervous system in the long-term developing neuroinflammation alongside other complications like brain atrophy, neuropathies etc.(Kim et al. 2021; Ramos-Rodriguez et al. 2014; Wrighten et al. 2009).

### Mechanism of action of hypoglycaemic agents, role in neuroinflammation and adverse effects

**Fig. 1:**
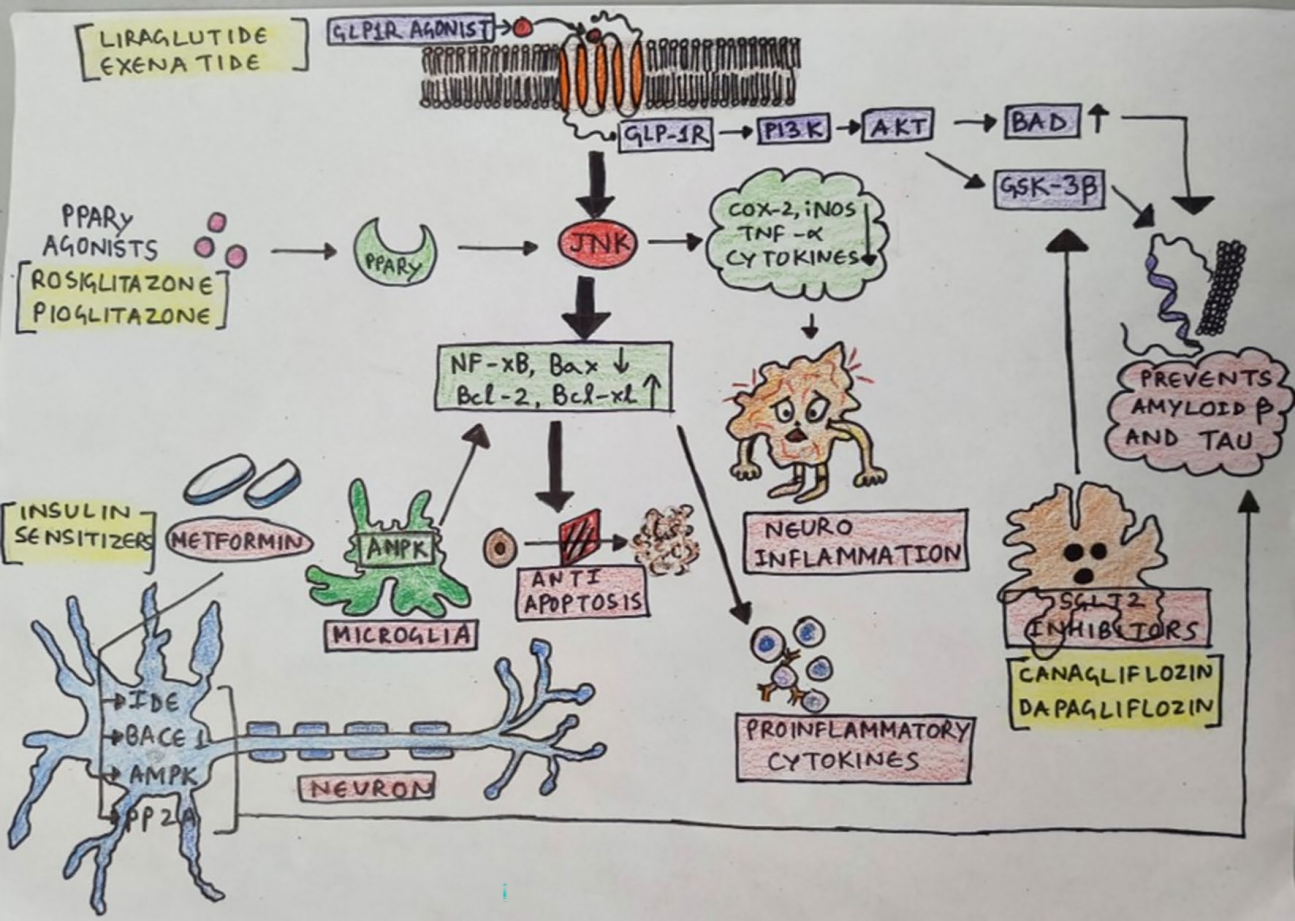
Diagrammatic representation of the molecular basis and mechanism of action of commonly prescribed antidiabetic drugs

**Fig. 2:**
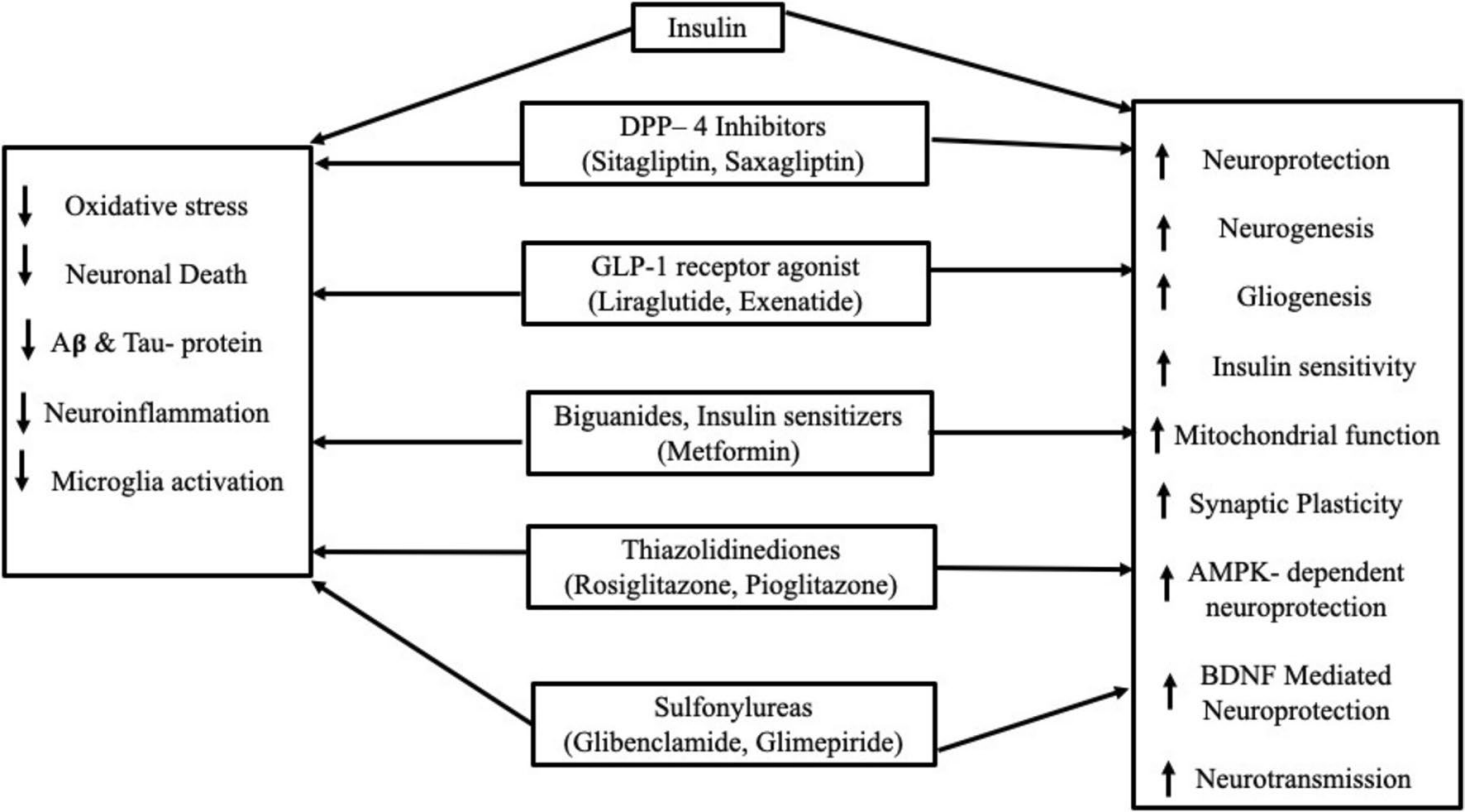
The beneficial effects of antidiabetics in the neuroprotection

The oral hypoglycaemics include sulfonylureas, biguanides, cotransporter inhibitors of sodium-glucose, inhibitors of DP-4, glucagon like peptide–1 agonist, thiazolidinedione, meglitinide, inhibitors of α-glucosidase, and glucose-dependent insulinotropic polypeptide agonists, varieties of insulins, amylinomimetics, bile acid sequestrants, dopamine agonists (Yang et al. 2023; Corcoran and Jacobs 2023; Di Magno et al. 2022; Guo et al. 2020; Wilcox 2020; Chen et al. 2018; Chaudhury et al. 2017; Standards of Medical Care in Diabetes 2016; Chamberlain et al. 2016). Table 1 summarises the mechanism of action and adverse effects of hypoglycaemic drugs, which are prescribed for the diabetes mellitus. The molecular basis and mechanism of action of commonly prescribed drugs are represented in Fig. 1 and Table 2. The beneficial effects of antidiabetics in the neuroprotection are highlighted in Fig. 2.

**Table 1:** Hypoglycaemic with their type and mechanism of action

class	drugs	mechanism of action and adverse effects
Biguanides	Metformin	They lower the blood glucose by acting primarily on the liver and decreasing glucose production by hepatic gluconeogenesis. Further it enhances the glucose absorption by skeletal muscle thus increasing peripheral insulin sensitivity, and decreasing insulin resistance. They are generally considered safe as they do not cause hypoglycaemia. Their absorption is from the intestines, and are eliminated rapidly through the kidneys. Therefore, caution is indicated in renal damage
Sodium-glucose cotransporter 2 (SGLT2) Inhibitor	Empagliflozin, Ertugliflozin, Canagliflozin, Dapagliflozin,	These will increase the renal excretion of glucose and sodium by acting on the proximal convoluted tubules. The reabsorption of sodium and glucose is prevented by acting on the SGLT2. Due to this, the side effects of them include urinary tract infections, diabetic ketoacidosis etc
GLP-1 RA	Exenatide, Liraglutide, Lixisenatide, Dulaglutide, Exenatide, Semaglutide	They mimic the action of the hormones secreted in the small intestine. Thereby Stimulate Insulin Release: GLP-1 triggers and stimulates the insulin release from the pancreas Inhibition of secretion of glucagon Slow down Gastric Emptying which reduces the release of glucose from the food into the bloodstream Enhance Satiety
DPP-4 inhibitors	Alogliptin, Linagliptin, Sitagliptin, Saxagliptin,	They protect the functioning of pancreatic islet cell and increase insulin levels. Glucose homeostasis is regulated by the hormone incretin which increases insulin in circulation to counteract the glucagon levels. DPP-4 inhibitors are involved in prolonging the action of these hormones. The biologically active peptides like GLP-1 and GIP are degraded
Thiazolidinediones (TZDs)	Rosiglitazone Pioglitazone,	Thiazolidinediones increase the peripheral insulin sensitivity in and in the liver. They may preserve the beta cells of pancreas and activate the PPAR gamma. Demonstrated consistent anti-inflammatory effects and potential benefits in reducing cardiovascular risks, effective in improving glycaemic control
Sulfonylureas (2nd generation)	Glipizide, Glyburide, Glimepiride	Stimulates insulin secretion from pancreatic beta cells, potential anti-inflammatory effects through inhibition of NLRP3 inflammasome
Insulins	Slow acting, Rapid acting- Human Analogs	Provides exogenous insulin to lower blood glucose levels; may have secondary effects on inflammation via improved glycaemic control Highly effective for glycaemic control; unclear direct effects on neuroinflammation as compared to other agents. The adverse effects include risk of hypoglycaemia, weight gain, injection site reactions
Alpha glucosidase inhibitors	Acarbose, Miglitol	They delay the absorption of carbohydrates and avoid the postprandial glucose hike. Their adverse effect includes gastrointestinal disturbances and may require titration of the dose
Amylinomimetics	Pramlintide	These agents mimic endogenous amylin, the effects of which is delayed gastric emptying, which further decreases the postprandial glucagon release
Dopamine agonist	Bromocriptine	Quick-release of bromocriptine resets the elevated hypothalamic drive for hyperglycaemia by acting on the hypothalamic circadian neuronal activity
Bile Acid Sequestrants	Colesevelam	Binds bile acids in the intestine to lower cholesterol levels; may have indirect effects on glucose metabolism and inflammation. But they have limited evidence for glycaemic control but may improve lipid profiles, unclear role in neuroinflammation treatment. The adverse effects include gastrointestinal discomfort, constipation and potential malabsorption of nutrients

**Table 2:** Molecular basis and mechanism of action of commonly prescribed antidiabetic drugs

Type	Commonly prescribed drug	Mechanism of action, molecular basis
Insulin sensitizers	Metformin, thiazolidinediones (e.g. rosiglitazone)	Increases the insulin sensitivity at liver and myocytes, activation of AMPK pathway (Pai et al. 2009), reduces inflammatory markers such as TNF-α and IL-6
Insulin secretagogues	Sulfonylureas (e.g. glimepiride, glipizide), meglitinides (e.g. repaglinide)	Binds to ATP-sensitive potassium channels at the β-cells of pancreas and stimulates insulin release (Aldhahi et al. 2004)
GLP-1 receptor agonists	Exenatide, liraglutide	Activates GLP-1 receptors, increases the glucose dependent secretion of insulin, delays gastric emptying (Vaughan and Santiago-Delgado 2024), lowers inflammatory cytokines
DPP-4 inhibitors	Sitagliptin, saxagliptin	Prolonging the incretins action by inhibiting the DPP-4 enzyme (Vaughan and Santiago-Delgado 2024), which help regulate insulin and glucagon
SGLT-2 inhibitors	Dapagliflozin, empagliflozin	Increases the urine glucose excretion and prevents the reabsorption of glucose at the kidney (Freeman 2013; Avranas et al. 2018)
α-Glucosidase inhibitors	Acarbose, miglitol	Inhibition of intestinal α-glucosidase and delaying the absorption of carbohydrate
Glinides	Repaglinide, nateglinide	Stimulates insulin release by closing the ATP-sensitive potassium channels (Aldhahi et al. 2004)
Glimines	Imeglimin	Moderation of mitochondrial function, enhancement of insulin action and protection of cells from the oxidative stress (Kuznetsov et al. 2022)

The mechanisms, clinical effectiveness, and adverse effects of hypoglycaemics, when are already used in the treatment of neuroinflammation is summarised in Table 3. The long-term use of hypoglycaemic medications in nondiabetic patients may pose certain risks including the hypoglycaemia, cardiovascular adverse effects and other metabolic disturbances. Thiazolidinediones like rosiglitazone can have cardiovascular adverse effects (Ishmuratova et al. 2023; Pfützner et al. 2007). GLP 1 receptor agonists (liraglutide) are reported to increase the pulse rate and blood pressure. The DPP-4 inhibitors like saxagliptin may increase the risk of heart failure, because of their higher sympathetic activity and prolonged stimulation of β-adrenergic system (Ong and Sia 2025). It is advised that, they should be managed additionally and the impact of the medications on the central nervous system needs a thorough evaluation to avoid the unintended consequences.

**Table 3:** Mechanisms, clinical effectiveness, and adverse effects of hypoglycaemics in treating neuroinflammation

Hypoglycaemic medication	Mechanism of action	Benefit in neuroinflammation	Adverse effects
GLP-1 agonists (liraglutide, exenatide)	1. Mimics glucagon-like peptide-1 (GLP-1), enhancing insulin secretion and reducing glucagon 2. Crosses the blood–brain barrier, promotes neuroprotection and reduces the apoptosis 3. Reduces the appetite	Strong evidence of anti-inflammatory effects in neurological disorders. It reduces the microglial activation and neuroinflammatory markers. They may improve the cognitive function in diabetes and delay the progression of neurodegenerative diseases like AD (Hung and Lu 2024; Zhu et al. 2016)	Gastrointestinal side effects including nausea and vomiting, risk of pancreatitis and thyroid tumors, which are reported in animal studies
Thiazolidinediones (rosiglitazone, pioglitazone)	Activates PPARγ, leading to improved insulin sensitivity and reduction of inflammatory markers like CRP and IL-6	Attenuates inflammation in the central nervous system and promotes the survival of dopaminergic neurons in the nigrostriatal system after the diffuse brain injury (documented in the animal model studies)	Weight gain, fluid retention, increased risk of heart failure and bone fractures
Insulin sensitizers (metformin)	1. Activation of AMP-activated protein kinase (AMPK) 2. Improves glucose metabolism and mitochondrial function	Reduction in the levels of neuroinflammatory markers like TNF-α, IL-6, enhancement of neuroprotection and reduction in oxidative stress as observed in the animal model studies (Li et al. 2022). Shown to improve cognitive function in AD	Gastrointestinal upset including nausea, vomiting and diarrhoea, lactic acidosis, deficiency of vitamin B12
Sodium glucose cotransporter 2 inhibitors (canagliflozin, dapagliflozin)	Prevents glucose reabsorption in the kidneys (Gui et al. 2024; Hung and Lu 2024), promotes glycosuria Effective in improving glycaemic control and may offer cardiovascular protection	Reduces oxidative stress and exhibits anti-inflammatory effects, enhances autophagy, and inhibits apoptosis (Arab et al. 2023) Improvement in cognitive function in diabetic animal models by the reduction of accumulation of amyloid-beta and oxidative damage Emerging evidence for neuroprotective effects	Dehydration due to increased glucose excretion and urinary tract infections Genital infections, dehydration, hypotension, risk of diabetic ketoacidosis
DPP-4 inhibitors (sitagliptin, saxagliptin)	Increases incretin levels, enhances the insulin secretion and decreases the release of glucagon	Neuroprotection by reduction of oxidative stress and inflammation (ElGamal et al. 2023) Moderate effects on inflammation with some evidence for cognitive benefits	Headaches, gastrointestinal symptoms, nasopharyngitis, headache, and risk of pancreatitis
Sulfonylureas (glibenclamide, glimepiride)	1. Stimulates insulin secretion by binding to the sulfonylurea receptors on β-cells 2. Reduces systemic inflammation indirectly by improving glycaemic control	Limited direct evidence for reducing the neuroinflammation, but has potential role in reducing the systemic inflammation May have limited benefits for advanced stage of neurodegenerative disease (He et al. 2022; Song et al. 2017)	Hypoglycaemia, weight gain and potential cardiovascular risks
Glycoside hydrolases inhibitor (acarbose)	1. Delays carbohydrate absorption in the gastrointestinal system 2. Reduces the level of postprandial blood glucose	May reduce the risk of stroke by controlling the postprandial hyperglycaemia	Gastrointestinal issues like flatulence, diarrhoea, and abdominal pain (Patel 2016)

#### Specific molecular targets of antidiabetic drugs

Antidiabetic drugs work through various mechanisms to decrease the serum glucose profile, with each class of drugs targeting specific molecular pathways. Each of these molecular targets plays a role in managing different aspects of insulin sensitivity and glucose metabolism, contributing to the overall control of diabetes. Here are some key molecular targets of common antidiabetic drugs.

#### Insulin sensitizers (metformin)

These drugs improve the body’s response to insulin without increasing the insulin secretion. Metformin belongs to the group ‘biguanides’, mainly targets the AMP-activated protein kinase (AMPK). This enhances the glucose uptake in peripheral tissues and decreases the neoglucogenesis, the glucose production in the liver. It also affects the mitochondrial respiratory chain complex 1.

### Metformin

The repurposing of metformin as an anti-inflammatory drug prevents a promising aid for the therapeutic intervention in neurodegenerative disorders. Its ability to modulate the inflammatory pathways coupled with existing evidence in clinical and preclinical studies under crosses its potential role in enhancing brain health and mitigating cognitive decline associated with age-related diseases. Further research is needed to fully mitigate the mechanism of clinical implication of metformin and anti-inflammatory effect in its neurological context.

Metformin is associated with decreased levels of various inflammatory markers suggesting its ability to modulate the inflammatory response in the body. This induces inhibition of pathway involved in inflammation such as NLRP 3 inflammasomes, which plays a crucial role in neuroinflammation (Bai and Chen 2021; Poor et al. 2021). Neuroprotective effects in studies have shown that metformin can attenuate their inflammation and protect against the neurodynamic neuronal damage. For instance, it has been found to reduce amyloid beta burdened and tau pathology in animal model of AD indicating the potential of mitigating the key pathology, picture of neurodegeneration (Bai and Chen 2021; Lin et al. 2023). Metformin activates AMPK activated protein kinase that is a crucial regulator in cellular energy homeostasis. This activation leads to anti-inflammatory effect and improves insulin sensitivity, which can be beneficial to brain health. There are several observational studies, which have highlighted a correlation between metformin use and reduced risk in developing AD. Among the diabetic patients these studies suggest that long-term metformin therapy may confer protective effect against cognitive decline (Adem et al. 2024; Bai and Chen 2021). The preclinical animal model research support an impression that metformin can promote neurogenesis and upgrade the cognitive function by reducing the inflammation and oxidative stress in the brain. For example, metformin has been shown to enhance the memory in learning capabilities in models of AD potentially and other neurodegenerative disorders beyond AD. Anti-inflammatory properties of metformin may also be relevant for other conditions such as PD. The disease research indicates that it could help alleviate the neuroinflammatory processes, which are associated with these disorders.(Bai and Chen 2021; Lin et al. 2023).

#### Sulfonylureas (glibenclamide, glimepiride)

Sulfonylureas reduce the blood glucose level effectively. They are ATP sensitive potassium channels, which act on the beta cells of pancreas. Sulfonylureas attach to K-ATP channels and inhibit them leading to the depolarization of the beta cell membrane, which opens the voltage-gated calcium channels. The influx of calcium triggers the insulin release from the pancreatic beta cells.

#### Glibenclamide (glyburide)

Glibenclamide lowers the insulin by acting on the endocrine part of pancreas (Kim et al. 2016; Sola et al. 2015; Aquilante 2010). It was reported to have crossed the blood brain barrier (BBB), particularly after an ischemic episode in an animal model research (Angel and Bidet 1991; Ortega et al. 2012; Kim et al. 2021). It also effectively inhibits the NLRP3 inflammasome, suggesting that it may be used to treat neuroinflammation-related disorders (Su et al. 2017).

#### Gliquidone

Another drug, gliquidone is stated to decrease the cromakalim evoked potassium efflux, which was observed at the substantia nigra of midbrainin an animal model (Kim et al. 2016; Schmid-Antomarchi et al. 1990). Gliquidone lowers the insulin by acting on the beta cell’s ATP- K + channels (Kim et al. 2021). Depolarization occurs due to the inhibition of K^+^ efflux, and furthermore results in the calcium influx (De Wet and Proks 2015; Hong et al. 2016; Kim et al. 2021), which results in the insulin release (Kim et al. 2016; Yang and Berggren 2006). Studies on mice suggested that, gliquidone has properties to decrease the neuroinflammation and has potential benefits by targeting the NLRP3 inflammasome pathway, modulating the lipopolysaccharide induced micro and astroglial neuroinflammation by triggering the ERK/STAT3/NF—κB signalling pathways. (Kim et al. 2021). These results endorse that, sulphonylureas can be potential in the treatment of neuroinflammation.

### Enzyme inhibitors

#### Sodium glucose cotransporter 2 inhibitors (canagliflozin, dapagliflozin)

They target mainly the** s**odium glucose cotransporter 2 (SGLT2) at the proximal convoluted tubules and prevent the reabsorption of glucose in the kidneys, leading to increased glucose excretion in urine (glycosuria), which lowers blood glucose levels independently of insulin.

### Empagliflozin

Empagliflozin has been associated with cognitive improvement through various mechanisms, including inhibition of acetylcholinesterase (AChE), which is important for the neurotransmission. It increases the levels of brain derived neurotrophic factor (BDNF), that assists for the growth and survival of nerve cells and plasticity. It is neuroprotective as it plays a role in the neurovascular remodelling, which is essential in the cognitive function. It is also a SGLT2 inhibitor, which may offer protection against the ischemic brain damage by influencing the sodium influx through SGLT1 receptors. This is in connection to reperfusion damage, oedema, inflammation, neuronal death, size of lesion and cognitive decline. Empagliflozin, like other SGLT2 inhibitors, exhibits anti-inflammatory and antiatherosclerotic effects that can contribute to neuroprotection by dropping the oxidation in the brain.

### Dapagliflozin

It is a sodium-glucose cotransporter 2 inhibitor, which is involved in glycolysis, apoptosis, and neuroinflammation. It attenuates the apoptosis by lowering NF-κB, Cyt-c, lactate, and HK-II levels. It demonstrated antiglycolytic, anti-inflammatory, antiapoptotic, and autophagic properties. All of them have enhanced the behavioural results (Aroda and Ratner 2018; Birajdar et al. 2023; El-Sahar et al. 2020).

#### DPP-4 inhibitors (sitagliptin, saxagliptin)

DPP-4 inhibitors act on the dipeptidyl peptidase-4 enzyme and prevent the breakdown of incretin hormones (GLP 1 and GIP), thus prolonging their action. This leads to increased insulin and reduced glucagon secretions in a glucose dependent manner.

#### 11β hydroxysteroid dehydrogenase type 1 (11β-HSD1) inhibitor

The inhibitors of this enzyme may help to reduce the glucose production by lowering the glucocorticoid activity in the liver.

#### Glycoside hydrolases inhibitor (acarbose)

Acarbose is a glycoside hydrolases inhibitor, which has been explored for its potential effects in neuroinflammation, particularly due to its influence on the metabolic pathways that overlap with the inflammatory processes in the brain. Research shows that drugs like acarbose, which target metabolic dysfunctions such as insulin resistance, may help modulate neuroinflammatory responses as inflammation is tightly linked with metabolic disorders and neurodegenerative diseases like AD. This is mediated mainly by the key pathways such as the AMP- activated protein kinase (AMPK) and glycogen synthase kinase-3 (GSK-3), which play significant roles in regulating inflammation and neuronal health. According to literature, by modulating the blood sugar levels and preventing the postprandial hypoglycaemia, acarbose may indirectly reduce the neuroinflammatory response. In animal models, metabolic regulators like acarbose have also shown promise in reducing the inflammation-driven neurodegenerative processes, although more research is needed to solidify its role in this context (Singhal et al. 2014; Tricco et al. 2021).

#### Incretin-based therapies

These drugs enhance the action of incretin hormones, which increase insulin secretion in response to meals.

#### GLP 1 receptor agonists (liraglutide, exenatide)

They target the glucagon like peptide 1 (GLP 1) receptor, which copycat the action of endogenous GLP 1 in increasing the glucose driven insulin secretion, damping of glucagon secretion, delayed gastric emptying, and promoting the satiety centres, which decreases the food consumption and help in controlling the blood sugar level.

### Liraglutide

This GLP-1 receptor agonist has demonstrated encouraging effects in regulating hyperglycaemia and possibly lowering neuroinflammation. Patients who were obese and receiving liraglutide saw improvements in their glycaemic control and a decrease in neuroinflammatory markers in addition to weight loss (Candeias et al. 2015). The clinical trials also suggested that, this drug helps in weight reduction, inhibition of microglia, enhanced cognition. It is also antiapoptotic, anti-inflammatory, and neuroprotective in T2D, stroke, and AD cases (Candeias et al. 2015).

#### Thiazolidinediones (rosiglitazone, pioglitazone)

These drugs bind to peroxisome proliferator activated receptor-gamma (PPAR-γ) receptors, which regulate genes involved in glucose and lipid metabolism. This increases insulin sensitivity in adipose tissue, muscle, and liver by promoting glucose uptake and reducing insulin resistance. The PPARγ activation inhibits the inflammatory pathways such as NF kB and p38 mitogen activated protein kinase.

#### Pioglitazone

This exerts its beneficial effects on neuroinflammation through various mechanisms. It can suppress the activation of microglia by inhibiting the expression of inflammatory cytokines. Pioglitazone has been found to provide protection from amyloid-beta (Aβ)-induced neuroinflammation and amyloidogenesis. By targeting the glutamatergic and inflammatory pathways, pioglitazone can help reduce Aβ levels, which are associated with neuroinflammation and cognitive impairment (Aroda and Ratner 2018).

### Insulin secretagogues

#### Meglitinides (repaglinide, nateglinide)

These drugs stimulate the pancreas to release more insulin and act on the ATP sensitive potassium channels, which is similar to the sulfonylureas. They stimulate insulin release in response to the food intake, however they have a shorter duration of action.

#### Insulin mimetics

These drugs act by imitating the action of insulin or enhancing insulin effects.

#### Amylin analogues (pramlintide)

Amylin analogues target the amylin receptors. Pramlintide belongs to this classification of drugs, which acts on this amylin, which is also a hormone, which is secreted along with the insulin by the β-cells of pancreas. Amylin is also known to delay the gastric emptying time, decrease the glucagon levels immediately after food consumption, and promote the satiety, which is instrumental in decreasing the glucose levels.

#### Glucagon receptor antagonists (experimental)

They act through the glucagon receptor and block the glucagon receptors to reduce the effect of glucagon, such as stimulating the hepatic glucose production. This can help to lower the blood glucose levels, especially in people with diabetes mellitus who have inappropriately elevated glucagon levels.

#### Other experimental targets

The agonists of GPR40 (Free fatty acid receptor 1) receptor can enhance the insulin secretion in response to the elevated glucose levels. Agonists of GPR119 receptor are being investigated for their ability to stimulate the insulin and GLP-1 secretion.

### Insulin

The type of insulin is always based on the patient’s needs for controlling the blood glucose levels, throughout the day. Premixed and combinations of insulin are used for controlling the blood sugar spike, which happens after the meals. Lot of study is done and evidence is emerging, suggesting the fact that insulin resistance can affect the brain and is also associated with an increased risk of AD. Insulin’s role in regulating glucose metabolism in the brain is crucial for the cognitive function and also known to reduce insulin signaling in the brain. This may contribute to the development of amyloid plaques and neurodegenerative ions like in insulin resistance to AD pathology (Tricco et al., 2021b).

The exogenous insulin binds to the insulin receptor on the target cells like liver, muscle, and adipose tissue, initiating a cascade of events that promote the glucose uptake, storage, and utilization, while inhibiting the hepatic glucose production. Insulin in pharmacology focus on its role as a crucial hormone in glucose homeostasis and its therapeutic use in managing diabetes. The pharmacokinetics of insulin involves absorption of insulin which is typically administered subcutaneously, although it can be given intravenously or intramuscularly. In certain situation, like in diabetes ketoacidosis the absorption of insulin varies based on the injection site, abdomen, thigh or in the arm and the formulation and volume injected as well. After the absorption, insulin binds with the insulin receptors of cell membrane particularly in the liver, muscle and adipose tissue. The insulin has relatively shorter half-life in circulation, that is about four to six minutes. It is metabolized in the liver and kidney, where insulin degrading enzyme breaks it down and the kidney excretes inactive insulin breakdown products. Talking about the pharmacodynamics, insulin binds to the insulin receptors on the target cell triggering the activation of tyrosine kinase pathway. This leads to translocation of the glucose transporter. Few metabolic effects of insulin include enhancing the glucose uptake by the cell, promoting glucagon synthesis in the liver, muscle and inhibiting the glycogenolysis, which is the breakdown of glycogen into glucose. It promotes lipogenesis, which is fat storage, inhibits fat breakdown and contributing to energy storage. Insulin stimulates amino acid uptake and protein synthesis, especially in the muscles, while inhibiting the protein degradation.

#### Types of insulins

There are different types of insulin preparations, one among them is rapid acting insulins like Lispro, Actrapid and Aspart. Their onset is within 15 min, and peak at 1–2 h, the duration is 3–5 h and is taken around the meal times. The other one is short acting insulin, which is the regular insulin. Their onset is 30 min, peak at 2–4 h duration and lasts for 5–8 h. They are also taken during meal time and continuous infusion. There are intermediate acting insulins like the Glargine and Detemir, the onset of which is 1–2 h and no significant peak is there and duration is up to 24 h. These are used to provide basal insulin coverage throughout the day and we have the ultra-long-lasting insulins like the Degludec, the onset of which is in 30 to 90 min and lasts more than around 42 h. These provide steady basal levels. There are 2 more types of insulins like the premixed insulins, for example Humulin70/30, Novolog 70/30 and Humalog mix 75/25. The onset is based on the based components and the peak can vary based on the components again. The duration of action may be 10 to 16 h and this acts by combining the short and rapid acting insulins with the intermediate acting insulin to simplify the dosing and typically taken two-times in a day before meals. The other one is the inhaled insulin like the Afrezza, onset is about 12–15 min and the peak is around 30–60 min from the administration. The duration of action is for around 2–3 h, as it is an alternative delivered method via inhalation and used at the begging of the meals.

### Rationale for repurposing the drugs

There are only three FDA approved drugs, donepezil, rivastigmine, and galantamine available for the AD, which offer only symptomatic improvement and there is no intervention in the progression of disease. Hence, the invention of newer drugs is essential to increase the lifespan of the AD patients (Tripathi et al. 2019). However, the complexity of human brain makes it challenging to the medicinal chemist (Srivastava et al. 2019). Expert studies indicated that re- purposing drugs have proven to be a faster, safe with efficacy when compared to the traditional drug development methods. It also has higher success rates since it can bypass the initial development stages (Ashburn and Thor 2004; Krishnamurthy et al. 2022; Novack 2021). Repurposing of drugs specifically became popular during the Covid-19 pandemic, when the FDA granted emergency use authorization (EUA) for several repurposed drugs like remdesivir (Krishnamurthy et al. 2022).

The other benefits of repurposing drugs include:

#### Anti-inflammatory effect

It can help reduce neuroinflammation by targeting inflammatory pathways in the brain, these drugs can mitigate the detrimental effects of chronic inflammation on neuronal health (Birajdar et al. 2023).

#### Neuroprotection

Repurposed drugs with anti-inflammatory effects can provide neuroprotection by reducing inflammation-induced damage to neurons and supporting their survival. The patient outcomes can be improved by the preserving the integrity and neuronal function as these drugs are known to slow down the progression of neurodegenerative disorders. (Birajdar et al. 2023).

### Targeting the anabolic and catabolic pathways for neuroprotection

The rationale for targeting the neurotropic-synaptotropic anabolic pathways is to promote the neuronal survival and synaptic plasticity. Brain-derived neurotrophic factor (BDNF) enhances synaptic connectivity, long-term potentiation (LTP), and neuronal survival. Insulin-like growth factor-1 (IGF-1) plays a critical role in neuroprotection by reducing the oxidative stress and inflammation (Lu et al. 2013; Dyer et al. 2016). Activation of anabolic pathways helps counter synaptic dysfunction, which is a hallmark of AD and other neurodegenerative conditions (Selkoe and Hardy 2016). Inhibiting mitochondrial catabolic activity reduces the production of reactive oxygen species (ROS) and protects neurons (Lin and Beal 2006).

#### Modulation of immune responses

The immune response in the brain can me modulated by few of the repurposed drugs. Leading to a balanced immune environment which is less prone to excessive inflammation. By regulating the immune system’s activity in the central nervous system, the diabetic drugs help in miniating the neuroinflammatory homeostasis and hence preventing the neuroinflammation (Birajdar et al. 2023).

#### Reduction of neurotoxicity

It is very well known that the neuroinflammation is always linked to the release of certain molecules which are toxic to the neurodegenerative processes hence the reproposed drugs that can target the neuroinflammation can help reduce neurotoxicity and protect neurons from the further damage, thereby preventing the cognitive functions and neuronal health (Birajdar et al. 2023).

### Synergistic effects of combination of hypoglycaemics with antidementia drugs

Certain proposed hypoglycaemic drugs are known to have some amount of potential synergistic effects with existing currently available drugs for the neurodegenerative diseases. Hence their overall efficacy is enhanced, if they are combined. The clinicians can have a better control over the treatment of certain neurodegenerative disorders by combining different drugs and the complementary mechanisms of actions2, by this they can also achieve a better improvement in the patient outcomes in neurodegenerative diseases (Birajdar et al. 2023). According to the literature, combination therapy of hypoglycaemics with antidementia medications can offer synergistic effects, which can target multiple pathways simultaneously and offer better patient outcome. They work through several mechanisms, which include reduction in the oxidative stress, increase in the insulin sensitivity, and enhancement of release of the neurotransmitter.

It was reported that, metformin offered better memory performance over the time in cognitively normal patients, suggesting that it has significant cognitive benefits (Wu et al. 2020). Hypoglycaemics improved the motor function in Parkinson’s patients, which is considered as a disease-modifying therapy (Sobral et al. 2025). If fenofibrate is administered along with the pioglitazone, there will be improvement of the markers of oxidative stress and cellular morphology, which was observed in an insulin resistance-induced neurodegeneration animal model (Idowu et al. 2022). In diabetic patients, using drugs with multimodal mechanisms of action like cerebrolysin, may help in reduction of cognitive decline, overall brain health and vascular risk factors (Bornstein et al. 2014).

The preclinical animal model research and few clinical trials have shown synergism between the hypoglycaemics and neuroprotective agents. However, more well-designed clinical studies are essential to confirm their synergism and safety in humans (Barus et al. 2019; Schmitt et al. 2004). Further research is required to understand their interaction in the context of comorbidities, diabetes and neurodegeneration (Ikhsan et al. 2019; Bergantin 2019).

#### Clinical translation

Repurposing of the drugs with anti-inflammatory properties for neuroinflammation associated conditions allows for faster clinical translation compared to developing new drugs from scratch. These repurposed drugs have already undergone safety and efficacy testing for other clinical conditions, hence making them attractive candidates for repurposing in neuroinflammatory disorders (Birajdar et al. 2023).

### Emerging evidence and theoretical basis

Neuroinflammation can contribute to neuronal damage, synaptic dysfunction, and cognitive impairment, all of which are observed in diabetes as well (Ramos-Rodriguez et al. 2014). There have been clinical studies indicating that in addition to the glycaemic control provided by antidiabetics it also has anti – inflammatory properties and benefits cognition (Kim et al. 2021; Ramos-Rodriguez et al. 2014; Wrighten et al. 2009; Chatterjee and Mudher 2018). The brain's neuronal activity is altered by insulin insufficiency, compromised signalling, and insulin resistance. The IDE, or insulin degrading enzyme, is involved in the metabolism of amyloid β, its aggregation, and the accumulation of misfolded proteins in the cortical and hippocampus neural regions of the brain (Birajdar et al. 2023). According to the studies, antidiabetic medications improve cognitive impairment by lowering the side effects of insulin resistance, oxidative stress, serum lipid profile, neuroinflammation, and brain hypoglycaemia activity (Birajdar et al. 2023). Repurposing already-approved antidiabetic medications, such as insulin, metformin, thiazolidinediones, sodium-glucose co-transport-2 (SGCT-2) inhibitors, DPP-4 inhibitors, glucagon-like peptides (GLP-1), and insulin, may be a viable and effective approach to treat neuroinflammation.

In a study, post mortem tissues of the hypothalamus, from 32 T2DM patients using different antidiabetic therapies, and 17 nondiabetic controls were examined. In the infundibular nucleus (IFN), it concentrated on proopiomelanocortin (POMC) neurons, neuropeptide-Y (NPY) neurons, and two kinds of microglia (iba1 and TMEM119). The findings of which suggested that among the different drugs metformin administration is connected to certain alterations in hypothalamic neurons and microglial cells, and also type 2 diabetes is associated with unique alterations in these cells like increased production of proinflammatory factors, primarily generated by reactive microglia, the brain's resident innate immune cells. The results of these investigations showed that there was hypothalamic inflammation (Kalsbeek et al. 2020).

### Pathogenesis of neuroinflammation (Fig. 3)

**Fig. 3:**
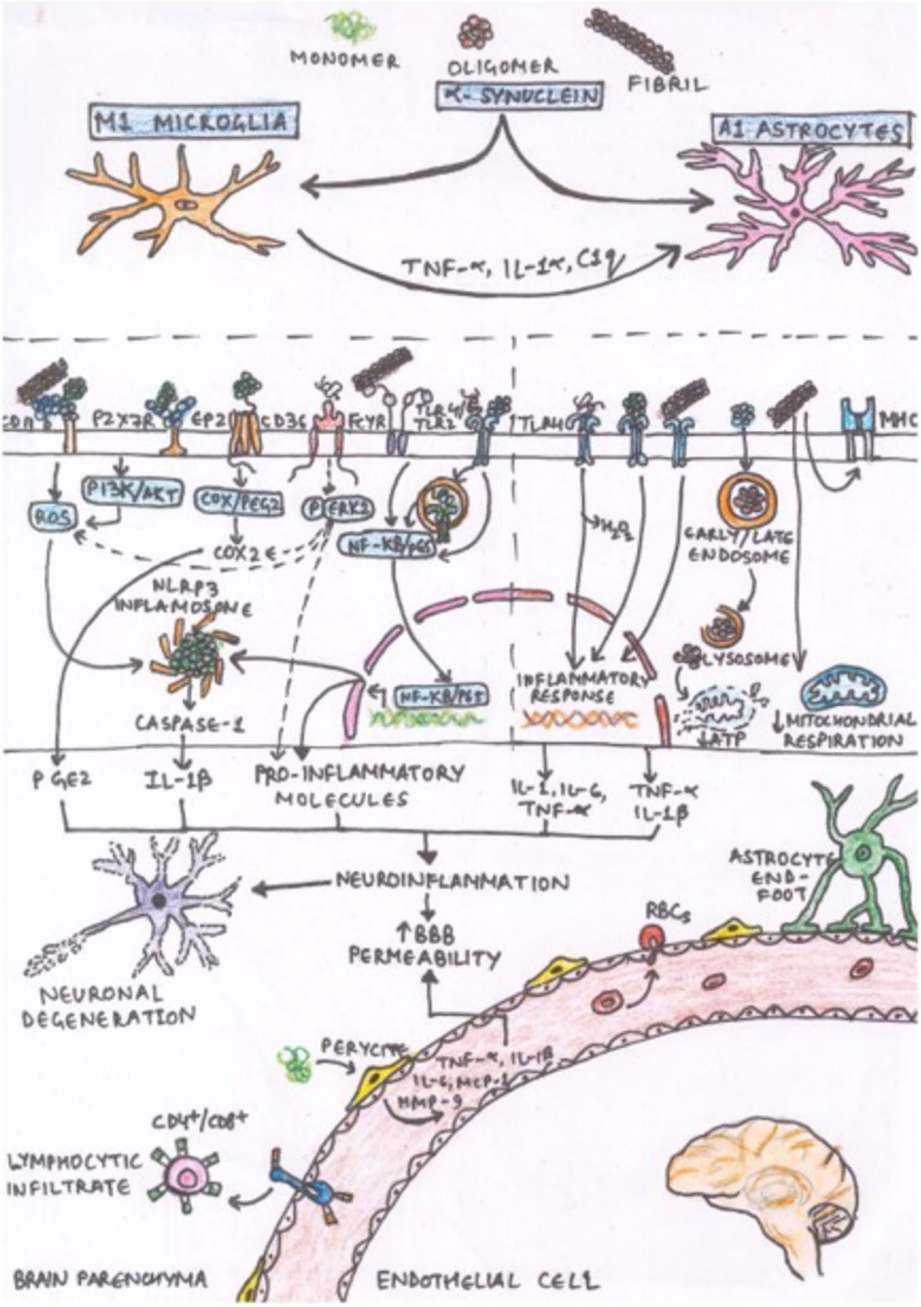
Diagrammatic representation of the neuroinflammation (reproduced from Delgado-Minjares et al. 2021)

Neuroinflammation involves activation of glial cells (microglia and astrocytes) and immune cells. The pathogenesis of neuroinflammation is multifaceted and involves various factors contributing to the inflammatory process.(Carson et al. 2006; Massa et al. 2006; Mattson et al. 1997), Here are key aspects of the pathogenesis of neuroinflammation:

#### Triggering events

Neuroinflammation can be triggered by various factors such as infections (viral, bacterial), traumatic brain injury, autoimmune reactions, chronic stress, environmental toxins, and neurodegenerative diseases (Carson et al. 2006; Massa et al. 2006; Mattson et al. 1997).

### Mechanism of neuroinflammation (Fig. 3)

The pathophysiology of neuroinflammation involves intricate interplay between systemic inflammatory responses and direct effects on the central nervous system. Inflammatory cytokine release, glial cell activation, BBB breakdown, and possible thromboembolic events are important mechanisms. (Different insults, such as TNF and mechanical injury, can cause neurons to express NF-κB. This neuronal stimulation is linked to the subsequent expression of INOS and SOD (superoxide dismutase) in the neurons (Mattson et al. 1997; Carson et al. 2006; Mattson et al. 1997; Rojo et al. 2004; Gorji 2022).

### Activation of glial cells (Fig. 3)

Microglia, the resident immune cells of the CNS, become activated in response to these triggers leading to the release of reactive oxygen species (ROS), chemokines, pro-inflammatory cytokines, contributing to the neuroinflammation (Carson et al. 2006; Massa et al. 2006; Mattson et al. 1997). Activation of microglial cells in the central nervous system can result in the release of pro-inflammatory cytokines, which worsen and spread neuroinflammation, which destroys healthy neurons and affects brain activity. Consequently, it is possible that activated microglia are important for neuroinflammatory processes (Hong et al. 2016). CNS-resident immune cells called microglia are triggered in reaction to brain damage (Hong et al. 2016; Stertz et al. 2013).

### Astrocyte activation (Fig. 3)

Astrocytes also become reactive in response to neurological insults. Reactive astrocytes release inflammatory mediators (Carson et al. 2006; Massa et al. 2006; Mattson et al. 1997). The involvement of astrocytes is known in a wide range of processes, including as immunological signalling, neurotransmitter recycling, blood–brain barrier (BBB) development and maintenance, ion and water homeostasis, and neuronal synaptogenesis control (Giovannoni and Quintana 2020). The nuclear factor kappa – light – chain – enhancer of activated B cells (NF-κB) pathway (Brambilla et al. 2005; Giovannoni and Quintana 2020), the calcineurin (CN) pathways (Furmanet al. 2014; Giovannoni et al. 2020), the mitogen-activated protein kinase (MAPK) pathway (Giovannoni and Quintana 2020; Roy Choudhury et al. 2014), and the Janus kinase/signal transducer and activator of transcription 3 (JAK/STAT3) pathway are some of the signalling pathways that both cause and modify astrocyte reactivity. Activation of NFκB (Mattson et al. 1997). Various stimuli like the cytokines (e.g., TNF-α, IL-1β), the pathogen-associated molecular patterns (PAMPs). The damage-associated molecular patterns (DAMPs), and the oxidative stress can activate NFκB in neurons (Mattson et al. 1997).

### Blood–brain barrier dysfunction (Fig. 4)

**Fig. 4:**
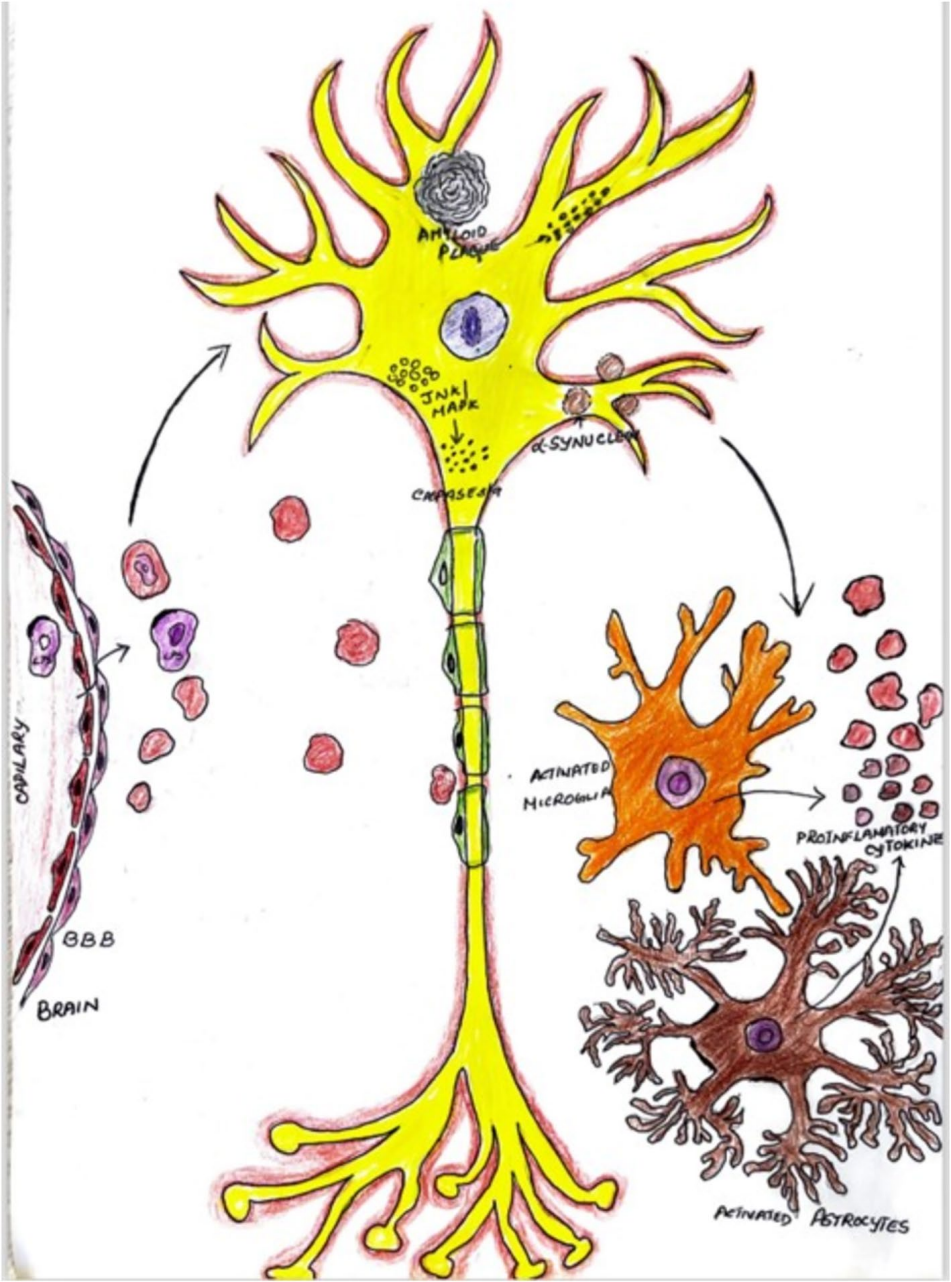
Schematic representation of process of neuroinflammation, showing the disruption of blood brain barrier (BBB) and entry of endotoxin like lipopolysaccharide into the brain microbiota and causing neuronal damage by the deposition of amyloid plaque, JNK MAPK and α-synuclein, leading to the activation of microglia and astrocytes resulting in cascade of inflammatory response

Infiltration of peripheral immune cells due to the disrupted blood–brain barrier (BBB) leads to increasing permeability which allows immune cells and inflammatory molecules to enter the brain, exacerbating neuroinflammation. There and leakage of the components in the blood into the brain by the disruption of the BBB which intern triggers the immune responses and hence contribute to neuroinflammatory processes (Carson et al. 2006; Massa et al. 2006; Mattson et al. 1997).

#### Cytokine release

The infiltrating immune cells release the TNF-α, IL-1beta, and IL-6 along with the activated glial cells, are known to further propagate the inflammatory cascade. This can lead to neuronal damage in various forms. Chemokines are known to attract immune cells to the site of inflammation in the brain, further propagating the ongoing inflammatory cascade (Carson et al. 2006; Massa et al. 2006; Mattson et al. 1997).

#### Oxidative stress

The nitrogen species and the reactive oxygen species generated during the neuroinflammation are also known to contribute to oxidative stress, causing damage to cellular components and also can cause neuronal damage and hence inflammation in the brain.(Carson et al. 2006; Massa et al. 2006; Mattson et al. 1997).

#### Neuronal damage

Chronic neuroinflammation can lead to neuronal dysfunction and death in various ways, hyper activation of glutamate receptors can lead to excitotoxicity, resulting in the neuronal injury and triggering inflammatory responses. Neuronal apoptosis, induced by various factors including inflammatory cytokines, can further exacerbate neuroinflammation and lead to neuronal apoptosis and degeneration and diseases like multiple sclerosis, AD and Parkinsons disease. (Carson et al. 2006; Massa et al. 2006; Mattson et al. 1997).

#### Chronic inflammation

Persistent neuroinflammation can result in chronic activation of immune responses, perpetuating the cycle of inflammation and neuronal damage is referred to as chronic inflammation (Carson et al. 2006; Mattson et al. 1997).

### Cellular and molecular pathways

#### Receptor activation

The activation of cell surface receptors like Toll-like receptors (TLRs), cytokine receptors, and growth factor receptors are known to initiates the signalling cascades leading to NFκB activation (Mattson et al. 1997).

#### Intracellular signalling

The downstream signalling pathways, including the canonical pathway involving IκB kinase (IKK) complex and the noncanonical pathway involving NFκB-inducing kinase (NIK), lead to the phosphorylation and also the degradation of inhibitory IκB proteins (Mattson et al. 1997).

#### Translocation to the nucleus

NFκB Complex Upon IκB degradation, NFκB dimers (e.g., p50/p65) are released and translocate to the nucleus (Mattson et al. 1997).

#### Gene transcription

In the nucleus, NFκB binds to specific DNA sequences in the promoter regions of target genes, leading to the transcription of pro-inflammatory cytokines, chemokines, and other inflammatory mediators (Mattson et al. 1997).

### Regulation of inflammatory response

#### Pro-inflammatory gene expression

NFκB activation in neurons results in the expression of inflammatory response genes, contributing to neuroinflammation(Mattson et al. 1997).

#### Feedback regulation

Negative feedback mechanisms, such as the expression of anti-inflammatory proteins like IκBα and A20, help regulate the duration and intensity of NFκB-mediated inflammatory responses (Mattson et al. 1997). The inhibition of NLRP3 inflammasome activation is a critical step in lowering the generation of IL-1β and the ensuing inflammation, and it occurs through both cellular and molecular mechanisms. For instance, glyburide specifically attacks this inflammasome(Su et al. 2017).

#### Evidence from preclinical studies—animal models of neuroinflammation

There have been numerous models of neuroinflammation which have been proposed over the years. To have a better understanding of the underlined mechanisms of neuroinflammation and the processes involved in neuroinflammation, its always essential to have a neuroinflammatory model. In order to understand the pathology of neurodegenerative disorders.

### Lipopolysaccharides (LPS) model

One of the most popular techniques for causing neuroinflammation in rodents is the LPS model. The gram negative bacterial especially E. coli on its outer cell wall or membrane has this component which is called the LPS. This LPS initiated an immune response that activates microglial and release pro-inflammatory cytokines like TNF-α, IL-1β and IL-6. The root of administration which is preferred is usually systemic that is intraperitoneal or through the intracerebroventricular administration straight into the brain. Now due to this the LPs model is useful for studying disease as it crosses the BBB (Fig. 4) by disrupting it and acting of the receptor cells in the causes the disassociation of the tight junction as a result of which the neuroinflammatory molecules enter the micro environment of brain hence useful on the study of disease like the AD and Parkinson’s disease because it can replicate a variety of behavioural dysfunction and cellular pathogenies, including synaptic deficits and cognitive impairments. (da Silva et al. 2024; Jung et al. 2023; Tamura et al. 2022).

### Polymeric acid—poly (1: C)

The systemic analogy of double standard RNA known as poly (1:C) also called the Model Poly (1:C). This is known to mimic the viral infection. Acts as an agonist for the toll-like receptors 3(TLR-3), which is part of the immune system responsible for recognizing the viral pathogens. Upon admiration this poly 1 stimulates the immune response which leads to the release of the proinflammatory cytokines such as interferons (IFNs), IL- 1β and the TNF- α this intern elicits activation of toll-like receptors (TLRs), specifically TLR4, by stimulating viral infections. This will result in the wide spreads neuroinflammation, mimicking the immune activation seen in viral infection and neurodegenerative conditions. Poly (1:c) induces a robust type 1 interferons response, mimicking viral infection rather than the bacterial-induced inflammation, like in the LPS model and hence make it more appropriate model for the study of the schizophrenia and autism. The consequences of virally induced neuroinflammation are investigated using this model. The root of administration is again may be systemic or brain infections are the two ways to administers poly (I:C) the results of this causes the upregulation of inflammation by increasing the inflammatory cytokines and microglial activation, which sheds light on the neuroinflammation brought on by the viral infections and its long-term impacts on cognition and behaviour. This model actually targets the main brain regions involved in neurodegeneration like the prefrontal cortex and hippocampus and few other regions in brain involving the memory and executive function (Ransohoff et al. 2015; Tamura et al. 2022).

#### Transgenic models

Human genes linked to AD are expressed in transgenic mouse models, such as the APPP/PS1 and 3XTg-AD mice. The accumulation of amyloid -beta (Aβ) plaque in these models demonstrate the generation of cytokines and microglial activation in response to Aβ deposition. This is employed in the investigation of the course of neuroinflammation and connection between neurodegeneration and cognitive decline (Ransohoff et al. 2015; Tamura et al. 2022).

#### Toxin-induced models

There are various toxins which are induced such as the 6-hydroxydopamine or kainic acid these can induce neuroinflammation by causing direct neuronal damage and subsequent inflammatory responses the root of administration is typically injecting into the specific brain regions the outcome of this models is very useful in the study of excitotoxicity and its role in neurodegenerative diseases particularly in the context of Parkinson’s disease (Ransohoff et al. 2015; Tamura et al. 2022).

#### Chronic social stress models

Chronic social stress, often induced by social defeat or isolation, can lead to neuroinflammation in the brain. This model mimics the effects of stress on the immune system, leading to increased levels of pro-inflammatory cytokines. As outcomes It is particularly relevant for studying the relationship between stress, neuroinflammation, and psychiatric disorders such as depression and anxiety (Bersano et al. 2023; Vizuete et al. 2022).

### Experimental autoimmune encephalomyelitis (EAE)

EAE is an animal model of multiple sclerosis, induced by immunization with myelin proteins. This model mimics the autoimmune aspects of neuroinflammation, with infiltration of immune cells into the CNS. As the outcomes: It is used to study the mechanisms of neuroinflammatory diseases and potential therapeutic interventions (Ransohoff et al. 2015; Vizuete et al. 2022).

These animal models provide invaluable insights into the mechanisms of neuroinflammation and its role in various neurological disorders. Each model has its strengths and limitations, and the choice of model often depends on the specific research questions being addressed. Understanding these models is crucial for developing effective therapeutic strategies for neuroinflammatory diseases. In vitro studies on neuroinflammation treated by antidiabetic drugs, the commonly used anti diabetic drug metformin has been demonstrated to reduce LPS-induced neuroinflammation in vitro. Metformin pretreatment considerably decreased the neuronal injury and apoptosis in a study involving the primary hippocampal neurons when compared with the LPS treated alone. Metformin functions by blocking the translocation of NF-κB p65 to the nucleus and reducing the expression of proinflammatory cytokines, such as TNF-α, IL-1β, and IL-6. In vitro, it was discovered that the antidiabetic medication gliquidone influences LPS-induced microglial neuroinflammatory responses. The gliquidone decreased the release of the inflammatory cytokines from the LPS-stimulated microglial cells and inhibited the activations of the NLRP3 inflammasome. These are component of innate immune system responsible for the release of inflammatory cytokines directly or indirectly. These results imply that gliquidone may be therapeutically useful for the disorders mediated by neuroinflammation. These in vitro investigations shed light on the ways in which antidiabetic medication to how these medications might be used again to treat neuroinflammatory neurogenerative diseases (Bai and Chen 2021; Michailidis et al. 2022; Vizuete et al. 2022; Zhang et al. 2021).

### Clinical evidence and trials

The utilization of antidiabetic medications including metformin, sitagliptin (DPP-4 inhibitors), pioglitazone and rosiglitazone (thiazolidinediones), dapagliflozin, empagliflozin (inhibitors of the sodium–glucose co transporter) and exenatide, liraglutide (glucagon- like peptides) demonstrated efficacy in preventing neurodegeneration. One such medication is the metformin. Biguanide is an oral drug used as first line of management for the noninsulin dependent diabetes mellitus. Adenosine monophosphate-activated receptors(mTOR) and the hepatic protein kinase (AMPK) mechanisms that initiate glucose absorption and then inhibits the comple-1 step involved in the gluconeogenesis the activity of mitochondrial enzymes. These medications can lessen the oxidative stress in both neuroinflammation and in neurodegeneration. The outcome against neurological illnesses was noted in the case of the various antidiabetic medications among the various models of the disease, ex-vivo research and in vitro studies (Birajdar et al. 2023). There has been certain reported that metformin has anti-inflammatory properties. It is unclear, nevertheless, if metformin causes the neuroprotection and antineuroinflammation in APP/PS1 mice. Here, we demonstrated that metformin improved neurogenesis in APP/PS1 mice while attenuating the deficit in spatial memory and hippocampal cell loss. Furthermore, the treatment of metformin reduced the amount of amyloid-β(Aβ) plaque levels as well as chronic neuroinflammation in the cortex and hippocampus, which included activated microglia, astrocytes, and pro-inflammatory mediators. Subsequent research revealed that metformin therapy increased brain AMPK activation. In the meantime, metformin significantly inhibited the activation of mTOR, S6K, P65 NF-κB, and decreased the productions of Bace1 protein. Our findings indicate that metformin can promote neurogenesis and have a neuroprotective effect on APP/PS1 mice by regulating the AMPK/mTOR/S6K/Bace1 and AMPK/P65 NF-κB pathways in the hippocampus. This may help to improve neurological deficits. Metformin can also functionally recover from memory deficits. Clinical evidence shows that therapies targeting the NLRP3 inflammasome might be a novel strategy for treating depression. Anakinra, an IL-1 receptor antagonist, improved glycaemia and β-cell function in patients suffering from the type 2 diabetes (T2D).

Hence, antidiabetic drugs can be used in the treatment of certain neuroinflammatory conditions with effective potentials (Su et al. 2017). Hypoglycaemic agent’s oral administration in neuroinflammation The NLRP3 inflammasome and its consequent generations of IL −1 β are involved in thein the common pathways between neuroinflammation and diabetes. Diabetes and depression are linked to innate immune overreaction and chronic hyperactivation of the HPA axis through immunological responses, glucose corticoids can actuate the NLRP inflammasome and cause insulin resistance (Su et al. 2017). In type 2 diabetes (T2D), chronic inflammatory responses in the brain play a detailed role in mediating injury. This occurs via the microglial and other immune cells activation leading to the production of neurotoxic quantity of free radicals and proinflammatory cytokines. This will eventually lead to brain disorders like diabetic neuropathy and encephalopathy, thereby increasing the chances of dementia. In addition, brain insulin resistance is proposed as a potential link between T2D and AD. Evidence includes decreased insulin receptor expression in AD patients; brains, accumulation of hyperphosphorylated tau, and increased amyloid-beta (Aβ) accumulation. The brain inflammation that that persists over time is a major mediator of damage in type 2 diabetes (T2D). by releasing neurotoxic levels of proinflammatory cytokines and free radicals activated microglial ad immune cells cause this to happen. The likelihood of dementia increases a result of this process, which also causes neurodegeneration ad brain disorders such as diabetes encephalopathy and neuropathy. AD and type 2 diabetes (T2D) also may be related to brain insulin kind of resistance. According to (EM et al., 2015) (Candeias et al. 2015). There is evidence of the amyloid beta (A β) accumulation, with reduced insulin receptors expression, and hyperphosphorylated tau accumulation in the brain of AD patients. There are certain pathways which are shared and contributed to by both type 2diabetes mellites(T2DM) and AD in its development and the progression Here are the key shared pathways.

### Biomarkers for monitoring the neuroinflammation and treatment efficacy

Repurposing antidiabetic drugs for neuroinflammation requires well-designed clinical trials and reliable biomarkers to examine the efficacy of drug. Biomarkers like TREM2, IL-1β, MCP-1, IL-6, TNF-α receptor complexes, TGF-β, and YKL-40 can be utilized to evaluate the progress of neuroinflammation and treatment efficacy (Tran et al. 2024; Hampel et al. 2020). However, further sophisticated outcome measures are necessary to evaluate the efficacy of antidiabetic drugs in treating neuroinflammation. Blood-based biomarkers like GFAP and sTREM2, imaging markers like central vein sign and paramagnetic rim lesions in the nuclear magnetic resonance, PET tracers targeting TSPO and MAO-B can predict the progression of neuroinflammation (Roveta et al. 2024; Lista et al. 2024).

#### Insulin resistance

The insulin resistance, is observed and exhibited in both the conditions T2DM and AD, which is also known to affects not only the function but also the structure of brain. Impaired insulin signalling may lead to neuroinflammation along with the cognitive decline, because insulin plays a imperative role in metabolism in brain and neuronal health (Zhu et al. 2023; Sivalingam et al. 2022).

#### Oxidative stress

Oxidative stress can be the consequence of chronic hypoglycaemia as in case of diabetes, as it is in the case of neuroinflammation. The inflammatory pathways get triggered by this oxidative stress, causing the neuronal injury and contributing to conditions like diabetic neuropathy (Sivalingam et al. 2022; Gunter 2005). Inflammatory Cytokines T2D and neuroinflammation are both conditions which are both characterized by the raised levels of pro-inflammatory cytokines. Hence the neuroinflammation can lead to neuronal damage and cognitive deficits, linking these two diseases.

#### Amyloid-beta accumulation

One of the hallmarks of AD that is the amyloid-beta, is accumulated and causes the metabolic dysfunction in the condition of diabetes A. This accumulation is associated with neuroinflammation and cognitive decline. (Sivalingam et al. 2022; Tupas et al. 2020). Recent studies have demonstrated few of the common genetic factors and pathways that link T2DM and AD, suggesting that alterations in gene expression and methylation may be influenced by both these diseases. Which includes pathways related to neuroactive ligands and receptor interactions (Tupas et al. 2020; Guzmán and Gurrola-Díaz, 2021).

### Impact outcome of drugs based on the patient variability like genetic variation

The recent studies have explored the capability of hypoglycaemic medications in managing the neurodegeneration, which suggest that certain antidiabetic drugs may offer neuroprotective benefits beyond their glucose lowering effects. However, individual differences including the genetic variation and disease stage, significantly influence the efficacy of them in managing the neurodegenerative conditions. Genetic differences can have impact, on how patients respond to these medications. For instance, variation in the genes related to the drug targets can alter the therapeutic outcomes. According to the literature, the genetic variation in the sulfonylurea target genes like ABCC8 were linked with a lower risk of AD in the diabetics, which recommends that genetics plays a task in the treatment efficacy (Tang et al. 2022; Song et al. 2017). Early administration of hypoglycaemic agent in a patient with neurodegeneration might yield better results in comparison to its administration during the advanced stages. This is because of the varying degree of neuronal damage and disease pathology. For example, the efficiency of antidiabetic drugs in enhancing the cognitive function may differ based on the rigorousness of the illness (Wang et al. 2023). CytoHubba's maximal clique centrality method has significant impact in the identification and prioritization of hub genes by leveraging the network centrality. Other methods like degree, maximum neighbourhood component, and Fisher score offer valuable alternatives. However, maximal clique centrality offers a higher precision in predicting the essential genes, making it a robust tool in bioinformatics analyses (Chin et al. 2014). The hub genes are neuroprotective in the cerebellum through several mechanisms, which include promotion of neurodevelopment, regulation of neurotransmission, maintenance of calcium homeostasis, and enhancement of stress responses. However, these mechanisms differ in their neuroprotective functions based on the specific gene and the stage and type of neurodegenerative condition. The response to antidiabetic agents can vary widely among the individuals as it is persuaded by several circumstances such as disease stage, genetic makeup, and comorbidities. This variability complicates the standardization of treatment protocol for the neurodegenerative conditions (Koshatwar et al. 2023). These descriptions suggest that, implementing the hypoglycaemic medication to treat the neurodegenerative diseases face several challenges. The neurodegenerative disease are also heterogenous as the underlying mechanisms are complex, which makes it difficult to predict, which patients benefit from which hypoglycaemic medication. This necessitates personalized treatment approaches for each patient (Wang et al. 2017).

The research on the use of SGLT2 inhibitors have shown that the possibility of dementia and Parkinson’s disease decreases in diabetics. However, additional studies are needed to approve these results in the nondiabetic population (Kim et al. 2024). While preclinical studies are promising about the usage of hypoglycaemics in the neurodegenerative illness, the success in clinical trials are limited. It is suggested that more exploration is necessary to establish the welfare and effectiveness of these medications in various stages of neurodegenerative diseases.

### Region specific neuroprotective response and designing of drug

Neurons in the hippocampus and cerebral cortex are more prone to excitotoxicity and inflammation due to higher glutamate receptor density and metabolic activity (Coyle and Puttfarcken 1993). Cerebellum has lower synaptic density and metabolic demand compared to regions like the hippocampus or cerebral cortex, which makes broad application of its mechanisms challenging (Mattson and Magnus 2006). It has higher baseline levels of antioxidant enzymes like superoxide dismutase (SOD) and glutathione peroxidase, which are not easily replicated in other brain regions (Fiskum et al. 1999). Inhibition of mitochondrial activity in energy-demanding regions can worsen ATP deficits, impairing the neuronal function and survival (Swerdlow et al. 2014). This is the potential limitations of pharmacologically mimicking the cerebellum's endogenous neuroprotective response in other brain regions, given its unique cytometabolic characteristics. The drugs designed for specific brain regions or cell types could avoid off-target effects. For instance, nanoparticles carrying neurotrophic factors could target the hippocampus selectively (Kreuter 2014, 2001). Enhancing glycolysis or supplementing ketones could replicate cerebellar metabolic flexibility in other regions (Cunnane et al. 2020).

### Functional analysis of cytometabolic processes and therapeutic strategies

Functional analyses of cytometabolic processes can provide critical insights into the shared and distinct pathological mechanisms underlying various neurodegenerative disorders beyond AD, such as Parkinsonism (PD), Huntington’s chorea (HD), and amyotrophic lateral sclerosis (ALS). Mitochondrial dysfunction is a hallmark of these diseases, contributing to oxidative stress, impaired energy production, and neuronal vulnerability. This progressively led to an autophagic process, known as mitophagy, which further worsens this disease (Rai et al. 2020). For example, Complex I dysfunction and defective mitophagy are central to PD, while disrupted calcium homeostasis and mitochondrial dynamics are prominent in HD and ALS. Cerebellum can employ multiple compensatory mechanisms to maintain energy levels despite the downregulation of genes linked to the mitochondrial electron transport chain. These mechanisms include support from glial cells, redox buffering circuits, cosubstrate compensation, efficient mitochondrial trafficking, the action of uncoupling proteins, and hormonal regulation (Sheng 2017).

Understanding the cerebellum’s resilience to oxidative stress and metabolic dysregulation could inspire novel neuroprotective strategies, such as targeting mitochondrial health with coenzyme Q10 or mitochondrial-targeted antioxidants like MitoQ, and enhancing mitophagy via PINK1/parkin activation. Additionally, leveraging the cerebellum’s metabolic flexibility could inform therapies such as ketogenic diets or glycolytic enhancers to combat energy deficits. Enhancing antioxidant defenses, using superoxide dismutase mimetics, or modulating neurotrophic pathways like BDNF and IGF-1 could further support synaptic repair and neuronal survival across these disorders. Region-specific approaches are critical, as dopaminergic neurons in the substantia nigra (PD) may require targeted mitochondrial and oxidative stress interventions, while cortical neurons in HD may benefit from therapies enhancing proteostasis and energy metabolism. Such cytometabolic insights offer the potential for precision medicine strategies tailored to specific brain regions and disease stages, advancing therapeutic development for neurodegenerative disorders (Surmeier et al. 2017; Stafstrom and Rho 2012; Gandhi and Abramov 2012; Exner et al. 2012; Nagahara and Tuszynski 2011)*.*

### Synaptogenesis and neurogenesis in neuroprotection

Synaptogenesis and neurogenesis, both are critical processes in maintaining and repairing the brain. However, they offer distinct advantages in the context of neuroprotection, particularly in the neurodegenerative diseases like AD. Synaptic dysfunction precedes the neuronal loss in many neurodegenerative diseases, making synaptogenesis a more immediate target for therapeutic intervention (Verstraelen et al. 2018; Nieland et al. 2014). Synaptogenesis offers several advantages over neurogenesis in neuroprotection by focusing on restoring synaptic function and connectivity. The therapies can potentially provide immediate cognitive benefits and slow disease progression. Hence, understanding and leveraging the molecular mechanisms of synaptogenesis will be crucial in developing effective targeted therapies for dementia. Therapies aimed at enhancing the synaptogenesis can potentially stabilize or reverse the early cognitive decline by addressing the synaptic deficits (Lu et al. 2013; Verstraelen et al. 2018).

Synaptogenesis plays a vital role in maintaining the neuronal integrity, preventing synaptic loss, and supporting neuroplasticity, which are the key factors implicated in neurodegenerative disorders like AD, PD and HD. It is hypothesised that, pharmacologically enhancing the synaptogenesis in other brain regions could replicate the cerebellum's neuroprotective mechanisms. This has been supported by studies highlighting the cerebellum's intrinsic resistance to neurodegeneration, attributed to its robust synaptic plasticity and efficient metabolic regulation.

### Limitations of present-day research

Each of the oral hypoglycaemic have its own side effects, which needs to be considered. The response to antidiabetic agent in the neurodegenerative diseases might vary individually. Various factors like stage of the disease, genetic variations and comorbidities may play a role (Koshatwar et al. 2023). Proper monitoring, assessments of the treatment responses, and vigilant management of the protocol are essential in managing the neurodegenerative diseases. These guidelines can help in decision making and adjust as needed (Santiago and Potashkin 2023).

### Future research direction

Future implication of the present study includes performing human clinical trials and this requires a proper patient selection like identifying patients in the prodromic stages of neuroinflammation or individuals at a higher risk (Kapaki et al. 2022). It is understood that, hypoglycaemic medications can offer promise for treating the neurodegenerative diseases. However, patient variability and disease heterogeneity can present with significant challenges. It is reported that, personalized approaches for medicines by considering the genetic profile and the stage of disease are essential for the better treatment outcomes. Further research and clinical trials will be crucial in addressing these challenges and establishing the effective therapeutic strategies. Combining antidiabetic agents with other medicines of neurodegenerative diseases can be neuroprotective and offer better outcome. The combination therapy, for example hypoglycaemics along with an adjuvant like neuroprotective agents or antioxidants can be beneficial in using these drugs for the neuroinflammation (Tran et al. 2024). The combined regime can also provide synergistic effect, for example, an antiamyloid or anti-tau drug can be added (Koshatwar et al. 2023). The apparent molecular basis of the therapeutic intercession can be better outlined with the network pharmacology (Ramakrishna et al. 2024).

Administration of neuroprotective agents, utilization of growth factors, targeting the synaptic plasticity, exploring the naturally available compounds, precision autophagy modulation, and understanding the cerebellar resistance can address the challenges of reproducing the cerebellum's naturally low off-target effects in pharmacological interventions for AD. It is aimed to enhance the therapeutic efficacy while minimizing the adverse effects and improving the patient outcomes. Understanding the region-specific differences in mitochondrial function and gene expression can provide valuable insights into developing targeted therapies for neurodegenerative diseases. The discovery of genes uniquely upregulated or downregulated in the cerebellum offers valuable insights into the mechanisms of selective neuronal vulnerability in AD. This knowledge can inform the development of targeted therapies and improve our understanding of the disease's progression and resistance patterns.

The novelty of this review article is that, it provides a comprehensive knowledge of the larger classification of antidiabetics, which can be used to treat the neurodegenerative diseases. The potential side effects of the hypoglycaemic agents in these older patients, particularly the cardiovascular adverse effects are described. This study also addresses how insulin sensitizers have influence over the neuroinflammatory pathways and it bridges the knowledge gap between the metabolic diseases like diabetes mellitus and neurodegeneration.

## Conclusion

The interaction between diabetes and neuroinflammation highlights the significance of metabolic health in neurodegenerative diseases. Understanding these shared pathways can inform strategies for prevention and treatment, potentially targeting both conditions simultaneously.

## References

[CR1] Adem MA, Decourt B, Sabbagh MN (2024) Pharmacological approaches using diabetic drugs repurposed for Alzheimer’s disease. Biomedicines 12:99 38255204 10.3390/biomedicines12010099 PMC10813018

[CR2] Aldhahi W, Armstrong J, Bouche C, Carr RD, Moses A, Goldfine AB (2004) Beta-cell insulin secretory response to oral hypoglycemic agents is blunted in humans in vivo during moderate hypoglycemia. J Clin Endocrinol Metab 89(9):4553–4557 15356061 10.1210/jc.2004-0266

[CR3] Angel I, Bidet S (1991) The binding site for [3H]glibenclamide in the rat cerebral cortex does not recognize K-channel agonists or antagonists other than sulphonylureas. Fundam Clin Pharmacol 5:107–115 1649112 10.1111/j.1472-8206.1991.tb00704.x

[CR4] Aquilante CL (2010) Sulfonylurea pharmacogenomics in type 2 diabetes: the influence of drug target and diabetes risk polymorphisms. Expert Rev Cardiovasc Ther 8:359–372 20222815 10.1586/erc.09.154 PMC2860269

[CR5] Arab HH, Eid AH, Alsufyani SE, Ashour AM, El-Sheikh AAK, Darwish HW, Sabry FM (2023) Targeting autophagy, apoptosis, and oxidative perturbations with dapagliflozin mitigates cadmium-induced cognitive dysfunction in rats. Biomedicines 11(11):3000 38002000 10.3390/biomedicines11113000 PMC10669515

[CR6] Aroda VR, Ratner RE (2018) Metformin and type 2 diabetes prevention. Diabetes Spectr 31:336 30510389 10.2337/ds18-0020 PMC6243218

[CR7] Ashayeri Ahmadabad R, Khaleghi Ghadiri M, Gorji A (2020) The role of Toll-like receptor signaling pathways in cerebrovascular disorders: the impact of spreading depolarization. J Neuroinflammation. 10.1186/s12974-020-01785-6 32264928 10.1186/s12974-020-01785-6 PMC7140571

[CR8] Ashburn TT, Thor KB (2004) Drug repositioning: identifying and developing new uses for existing drugs. Nat Rev Drug Discov 3:673–683 15286734 10.1038/nrd1468

[CR9] Avranas K, Imprialos K, Stavropoulos K, Lales G, Manafis A, Skalkou A, Kihm L (2018) Sodium-glucose cotransporter 2 inhibitors: glucose lowering against other hypoglycemic agents. Cardiovasc Hematol Disord Drug Targets 18(2):94–103 29412124 10.2174/1871529X18666180206160838

[CR10] Bai B, Chen H (2021) Metformin: a novel weapon against inflammation. Front Pharmacol 12:622262 33584319 10.3389/fphar.2021.622262 PMC7880161

[CR11] Barus R, Béné J, Deguil J, Gautier S, Bordet R (2019) Drug interactions with dementia-related pathophysiological pathways worsen or prevent dementia. Br J Pharmacol 176(18):3413–3434 30714122 10.1111/bph.14607 PMC6715604

[CR12] Bergantin LB (2019) Hypertension, diabetes and neurodegenerative diseases: is there a clinical link through the Ca2+/cAMP signalling interaction? Curr Hypertens Rev 15(1):32–39 30117399 10.2174/1573402114666180817113242

[CR13] Bersano A, Engele J, Schäfer MKE (2023) Neuroinflammation and brain disease. BMC Neurol 23:1–3 37308838 10.1186/s12883-023-03252-0 PMC10258999

[CR14] Birajdar SV, Mazahir F, Alam MI, Kumar A, Yadav AK (2023) Repurposing and clinical attributes of antidiabetic drugs for the treatment of neurodegenerative disorders. Eur J Pharmacol. 10.1016/j.ejphar.2023.176117 37907134 10.1016/j.ejphar.2023.176117

[CR15] Bornstein NM, Brainin M, Guekht A, Skoog I, Korczyn AD (2014) Diabetes and the brain: issues and unmet needs. Neurol Sci 35(7):995–1001 24777546 10.1007/s10072-014-1797-2 PMC4064119

[CR16] Brambilla R, Bracchi-Ricard V, Hu WH, Frydel B, Bramwell A, Karmally S, Green EJ, Bethea JR (2005) Inhibition of astroglial nuclear factor kappaB reduces inflammation and improves functional recovery after spinal cord injury. J Exp Med 202:145–156 15998793 10.1084/jem.20041918 PMC2212896

[CR17] Bubber P, Haroutunian V, Fisch G, Blass JP, Gibson GE (2005) Mitochondrial abnormalities in Alzheimer brain: mechanistic implications. Ann Neurol 57(5):695–703 15852400 10.1002/ana.20474

[CR18] Candeias EM, Sebastião IC, Cardoso SM, Correia SC, Carvalho CI, Plácido AI, Santos MS, Oliveira CR, Moreira PI, Duarte AI (2015) Gut-brain connection: the neuroprotective effects of the anti-diabetic drug liraglutide. World J Diabetes 6(6):807–827 26131323 10.4239/wjd.v6.i6.807 PMC4478577

[CR19] Carson MJ, Cameron Thrash J, Walter B (2006) The cellular response in neuroinflammation: the role of leukocytes, microglia and astrocytes in neuronal death and survival. Clin Neurosci Res 6:237–245 19169437 10.1016/j.cnr.2006.09.004 PMC2630233

[CR20] Cenini G, Voos W (2019) Mitochondria as potential targets in Alzheimer disease therapy: an update. Front Pharmacol 10:465280 10.3389/fphar.2019.00902 PMC6716473 31507410

[CR21] Chamberlain JJ, Rhinehart AS, Shaefer CF, Neuman A (2016) Diagnosis and management of diabetes: Synopsis of the 2016 American Diabetes Association standards of medical care in diabetes. Ann Intern Med 164:542–552 26928912 10.7326/M15-3016

[CR22] Chatterjee S, Mudher A (2018) Alzheimer’s disease and type 2 diabetes: a critical assessment of the shared pathological traits. Front Neurosci. 10.3389/fnins.2018.00383 29950970 10.3389/fnins.2018.00383 PMC6008657

[CR23] Chaudhury A, Duvoor C, Reddy Dendi VS, Kraleti S, Chada A, Ravilla R, Marco A, Shekhawat NS, Montales MT, Kuriakose K, Sasapu A, Beebe A, Patil N, Musham CK, Lohani GP, Mirza W (2017) Clinical review of antidiabetic drugs: implications for type 2 diabetes mellitus management. Front Endocrinol (Lausanne) 8:224539 10.3389/fendo.2017.00006 PMC5256065 28167928

[CR24] Chen K, Kang D, Yu M, Zhang R, Zhang Y, Chen G, Mu Y (2018) Direct head-to-head comparison of glycaemic durability of dipeptidyl peptidase-4 inhibitors and sulphonylureas in patients with type 2 diabetes mellitus: a meta-analysis of long-term randomized controlled trials. Diabetes Obes Metab 20:1029 29095568 10.1111/dom.13147 PMC5873267

[CR25] Chin CH, Chen SH, Wu HH, Ho CW, Ko MT, Lin CY (2014) cytoHubba: identifying hub objects and sub-networks from complex interactome. BMC Syst Biol 8(Suppl 4):S11 25521941 10.1186/1752-0509-8-S4-S11 PMC4290687

[CR26] Corcoran C, Jacobs TF (2023). Metformin. Encyclopedia Biomed Gerontol. Volume 1–3 2, V2–424-V2–432

[CR27] Coyle JT, Puttfarcken P (1993) Oxidative stress, glutamate, and neurodegenerative disorders. Science 262(5134):689–695 7901908 10.1126/science.7901908

[CR28] Cunnane SC, Trushina E, Morland C, Prigione A, Casadesus G, Andrews ZB, Beal MF, Bergersen LH, Brinton RD, de la Monte S, Eckert A, Harvey J, Jeggo R, Jhamandas JH, Kann O, la Cour CM, Martin WF, Mithieux G, Moreira PI, Murphy MP, Nave KA, Nuriel T, Oliet SHR, Saudou F, Mattson MP, Swerdlow RH, Millan MJ (2020) Brain energy rescue: an emerging therapeutic concept for neurodegenerative disorders of ageing. Nat Rev Drug Discov 19(9):609–633 32709961 10.1038/s41573-020-0072-x PMC7948516

[CR29] da Silva AAF, Fiadeiro MB, Bernardino LI, Fonseca CSP, Baltazar GMF, Cristóvão ACB (2024) Lipopolysaccharide-induced animal models for neuroinflammation - an overview. J Neuroimmunol. 10.1016/j.jneuroim.2023.578273 38183948 10.1016/j.jneuroim.2023.578273

[CR30] De Wet H, Proks P (2015) Molecular action of sulphonylureas on KATP channels: a real partnership between drugs and nucleotides. Biochem Soc Trans 43:901–907 26517901 10.1042/BST20150096 PMC4613533

[CR31] Defronzo RA (2009) From the triumvirate to the ominous octet: a new paradigm for the treatment of type 2 diabetes mellitus. Diabetes 58:773–795 19336687 10.2337/db09-9028 PMC2661582

[CR32] Delgado-Minjares KM, Martinez-Fong D, Martínez-Dávila IA, Bañuelos C, Gutierrez-Castillo ME, Blanco-Alvarez VM, Cardenas-Aguayo MD, Luna-Muñoz J, Pacheco-Herrero M, Soto-Rojas LO (2021) Mechanistic insight from preclinical models of Parkinson’s disease could help redirect clinical trial efforts in GDNF therapy. Int J Mol Sci 22(21):11702 34769132 10.3390/ijms222111702 PMC8583859

[CR33] Di Magno L, Di Pastena F, Bordone R, Coni S, Canettieri G (2022) The mechanism of action of biguanides: new answers to a complex question. Cancers (Basel). 10.3390/cancers14133220 35804992 10.3390/cancers14133220 PMC9265089

[CR34] Dyer AH, Vahdatpour C, Sanfeliu A, Tropea D (2016) The role of insulin-like growth factor 1 (IGF-1) in brain development, maturation and neuroplasticity. Neuroscience 325:89–99 27038749 10.1016/j.neuroscience.2016.03.056

[CR35] ElGamal RZ, Tadros MG, Menze ET (2023) Linagliptin counteracts rotenone’s toxicity in non-diabetic rat model of Parkinson’s disease: insights into the neuroprotective roles of DJ-1, SIRT-1/Nrf-2 and implications of HIF1-α. Eur J Pharmacol 941:175498 36623635 10.1016/j.ejphar.2023.175498

[CR36] El-Sahar AE, Rastanawi AA, El-Yamany MF, Saad MA (2020) Dapagliflozin improves behavioral dysfunction of Huntington’s disease in rats via inhibiting apoptosis-related glycolysis. Life Sci 257:118076 32659371 10.1016/j.lfs.2020.118076

[CR37] Exner N, Lutz AK, Haass C, Winklhofer KF (2012) Mitochondrial dysfunction in Parkinson’s disease: molecular mechanisms and pathophysiological consequences. EMBO J 31(14):3038–3062 22735187 10.1038/emboj.2012.170 PMC3400019

[CR38] Feng B, Hu P, Chen J, Liu Q, Li X, Du Y (2015) Analysis of differentially expressed genes associated with Alzheimer’s disease based on bioinformatics methods. Am J Alzheimers Dis Other Demen 30(8):746–751 24965283 10.1177/1533317514537548 PMC10852745

[CR39] Fiskum G, Murphy AN, Beal MF (1999) Mitochondria in neurodegeneration: acute ischemia and chronic neurodegenerative diseases. J Cereb Blood Flow Metab 19(4):351–369 10197505 10.1097/00004647-199904000-00001

[CR40] Flamm AG, Żerko S, Zawadzka-Kazimierczuk A, Koźmiński W, Konrat R, Coudevylle N (2016) 1H, 15N, 13C resonance assignment of human GAP-43. Biomol NMR Assign 10:171–174 26748655 10.1007/s12104-015-9660-9 PMC4788685

[CR41] Freeman JS (2013) Review of insulin-dependent and insulin-independent agents for treating patients with type 2 diabetes mellitus and potential role for sodium-glucose co-transporter 2 inhibitors. Postgrad Med 125(3):214–226 23748522 10.3810/pgm.2013.05.2672

[CR42] Furman JL, Norris CM (2014) Calcineurin and glial signaling: neuroinflammation and beyond. J Neuroinflammation. 10.1186/s12974-014-0158-7 25199950 10.1186/s12974-014-0158-7 PMC4172899

[CR43] Gandhi S, Abramov AY (2012) Mechanism of oxidative stress in neurodegeneration. Oxid Med Cell Longev 2012:428010 22685618 10.1155/2012/428010 PMC3362933

[CR44] Gellersen HM, Guell X, Sami S (2021) Differential vulnerability of the cerebellum in healthy ageing and Alzheimer’s disease. Neuroimage Clin 30:102605 33735787 10.1016/j.nicl.2021.102605 PMC7974323

[CR45] Genter MB, Van Veldhoven PP, Jegga AG, Sakthivel B, Kong S, Stanley K, Witte DP, Ebert CL, Aronow BJ (2003) Microarray-based discovery of highly expressed olfactory mucosal genes: potential roles in the various functions of the olfactory system. Physiol Genomics 16:67–81 14570983 10.1152/physiolgenomics.00117.2003

[CR46] Gilhus NE, Deuschl G (2019) Neuroinflammation- a common thread in neurological disorders. Nat Rev Neurol 15(8):429–430 31263256 10.1038/s41582-019-0227-8

[CR47] Giovannoni F, Quintana FJ (2020) The role of astrocytes in CNS inflammation. Trends Immunol 41:805 32800705 10.1016/j.it.2020.07.007 PMC8284746

[CR48] Gorji A (2022) Neuroinflammation: the pathogenic mechanism of neurological disorders. Int J Mol Sci 23:23 10.3390/ijms23105744 PMC9147744 35628553

[CR49] Gui Z, Wang J, Zhang Y, Wan B, Ke Z, Ren Z, Yang X, Lei M, Guo X, Liu X, Ouyang C, Wu N, Chen Q (2024) Dapagliflozin improves diabetic cognitive impairment via indirectly modulating the mitochondria homeostasis of hippocampus in diabetic mice. BioFactors 50(1):145–160 37596888 10.1002/biof.1998

[CR147] Gunter M (2005) The mode of action of the antidiabetic drug glimepiride-beyond insulin secretion. Curr Med Chem - Immunol, Endocr Metab Agents 5(6):499–518

[CR50] Guo W, Li M, Dong Y, Zhou H, Zhang Z, Tian C, Qin R, Wang H, Shen Y, Du K, Zhao L, Fan H, Luo S, Hu D (2020) Diabetes is a risk factor for the progression and prognosis of COVID-19. Diabetes Metab Res Rev. 10.1002/dmrr.3319 32233013 10.1002/dmrr.3319 PMC7228407

[CR148] Guzmán TJ, Gurrola-Díaz CM (2021) Glucokinase activation as antidiabetic therapy: effect of nutraceuticals and phytochemicals on glucokinase gene expression and enzymatic activity. Arch Physiol Biochem 127(2):182–193 31210550 10.1080/13813455.2019.1627458

[CR51] Hampel H, Caraci F, Cuello AC, Caruso G, Nisticò R, Corbo M, Baldacci F, Toschi N, Garaci F, Chiesa PA, Verdooner SR, Akman-Anderson L, Hernández F, Ávila J, Emanuele E, Valenzuela PL, Lucía A, Watling M, Imbimbo BP, Vergallo A, Lista S (2020) A path toward precision medicine for neuroinflammatory mechanisms in Alzheimer’s disease. Front Immunol 11:456 32296418 10.3389/fimmu.2020.00456 PMC7137904

[CR52] Hampel H, Hardy J, Blennow K, Chen C, Perry G, Kim SH, Villemagne VL, Aisen P, Vendruscolo M, Iwatsubo T, Masters CL, Cho M, Lannfelt L, Cummings JL, Vergallo A (2021) The amyloid-β pathway in Alzheimer’s disease. Mol Psychiatry 26:5481–5503 34456336 10.1038/s41380-021-01249-0 PMC8758495

[CR53] He Y, Chang Y, Peng Y, Zhu J, Liu K, Chen J, Wu Y, Ji Z, Lin Z, Wang S, Gupta S, Zang N, Pan S, Huang K (2022) Glibenclamide directly prevents neuroinflammation by targeting SUR1-TRPM4-mediated NLRP3 inflammasome activation in microglia. Mol Neurobiol 59(10):6590–6607 35972671 10.1007/s12035-022-02998-x

[CR54] Holahan MR (2017) A shift from a pivotal to supporting role for the growth-associated protein (GAP-43) in the coordination of axonal structural and functional plasticity. Front Cell Neurosci 11:266 28912688 10.3389/fncel.2017.00266 PMC5583208

[CR55] Hong H, Kim BS, Im HI (2016) Pathophysiological role of neuroinflammation in neurodegenerative diseases and psychiatric disorders. Int Neurourol J 20:S2 27230456 10.5213/inj.1632604.302 PMC4895907

[CR56] Hu W, Lin X, Chen K (2015) Integrated analysis of differential gene expression profiles in hippocampi to identify candidate genes involved in Alzheimer’s disease. Mol Med Rep 12(5):6679–6687 26324066 10.3892/mmr.2015.4271 PMC4626122

[CR57] Hung CH, Lu LY (2024) New insights into the role of SGLT-2 inhibitors in the prevention of dementia. Neurol Int 16(6):1717–1730 39728750 10.3390/neurolint16060124 PMC11676485

[CR58] Idowu OK, Oluyomi OO, Faniyan OO, Dosumu OO, Akinola OB (2022) The synergistic ameliorative activity of peroxisome proliferator-activated receptor-alpha and gamma agonists, fenofibrate and pioglitazone, on hippocampal neurodegeneration in a rat model of insulin resistance. Ibrain 8(3):251–263 37786742 10.1002/ibra.12059 PMC10528802

[CR59] Ikhsan M, Palumbo A, Rose D, Zille M, Boltze J (2019) Neuronal stem cell and drug interactions: a systematic review and meta-analysis: concise review. Stem Cells Transl Med 8(11):1202–1211 31313515 10.1002/sctm.19-0020 PMC6811698

[CR60] Ishmuratova AN, Abramov MA, Kuznetsov KO, Ivanyuta MV, Shakirova ZF, Kitapova AI, Usmonov MD, Chernousova LM, Valeeva LI, Kuznetsova AY, Baislamov AS, Shaihetdinova AR, Mirgaliev AA, Orozberdiev ST, Yakupova KI (2023) The role of antidiabetic drugs in the treatment of Alzheimer’s disease: systematic review. Probl Endokrinol (Mosk) 69(5):73–83 37968954 10.14341/probl13183 PMC10680548

[CR61] Jung H, Lee D, You H, Lee M, Kim H, Cheong E, Um JW (2023) LPS induces microglial activation and GABAergic synaptic deficits in the hippocampus accompanied by prolonged cognitive impairment. Sci Rep 13(1):1–14 37085584 10.1038/s41598-023-32798-9 PMC10121592

[CR62] Kalsbeek MJT, Wolff SEC, Korpel NL, la Fleur SE, Romijn JA, Fliers E, Kalsbeek A, Swaab DF, Huitinga I, Hol EM, Yi CX (2020) The impact of antidiabetic treatment on human hypothalamic infundibular neurons and microglia. JCI Insight. 10.1172/jci.insight.133868 32814716 10.1172/jci.insight.133868 PMC7455135

[CR63] Kapaki E, Vakrakou AG, Boufidou F (2022) Novel CSF biomarkers tracking autoimmune inflammatory and neurodegenerative aspects of CNS diseases. Diagnostics (Basel) 13(1):73 36611365 10.3390/diagnostics13010073 PMC9818715

[CR64] Kim YK, Na KS, Myint AM, Leonard BE (2016) The role of pro-inflammatory cytokines in neuroinflammation, neurogenesis and the neuroendocrine system in major depression. Prog Neuropsychopharmacol Biol Psychiatry 64:277–284 26111720 10.1016/j.pnpbp.2015.06.008

[CR65] Kim J, Park JH, Shah K, Mitchell SJ, Cho K, Hoe HS (2021) The anti-diabetic drug gliquidone modulates lipopolysaccharide-mediated microglial neuroinflammatory responses by inhibiting the NLRP3 inflammasome. Front Aging Neurosci 13:754123 34776934 10.3389/fnagi.2021.754123 PMC8587901

[CR66] Kim HK, Biessels GJ, Yu MH, Hong N, Lee YH, Lee BW, Kang ES, Cha BS, Lee EJ, Lee M (2024) SGLT2 inhibitor use and risk of dementia and Parkinson disease among patients with type 2 diabetes. Neurology 103(8):e209805 39292986 10.1212/WNL.0000000000209805

[CR67] Koshatwar M, Acharya S, Prasad R, Lohakare T, Wanjari M, Taksande AB (2023) Exploring the potential of antidiabetic agents as therapeutic approaches for Alzheimer’s and Parkinson’s diseases: a comprehensive review. Cureus 15(9):e44763 37809189 10.7759/cureus.44763 PMC10556988

[CR68] Kowara R, Ménard M, Brown L, Chakravarthy B (2007) Co-localization and interaction of DPYSL3 and GAP43 in primary cortical neurons. Biochem Biophys Res Commun 363:190–193 17845802 10.1016/j.bbrc.2007.08.163

[CR69] Kreuter J (2001) Nanoparticulate systems for brain delivery of drugs. Adv Drug Deliv Rev 47(1):65–81 11251246 10.1016/s0169-409x(00)00122-8

[CR149] Kreuter J (2014) Drug delivery to the central nervous system by polymeric nanoparticles: What do we know? Adv Drug Deliv Rev 71:2–14 23981489 10.1016/j.addr.2013.08.008

[CR70] Krishnamurthy N, Grimshaw AA, Axson SA, Choe SH, Miller JE (2022) Drug repurposing: a systematic review on root causes, barriers and facilitators. BMC Health Serv Res 22:970 35906687 10.1186/s12913-022-08272-z PMC9336118

[CR71] Kuznetsov KO, Saetova AA, Mahmutova EI, Bobrik AG, Bobrik DV, Nagaev IR, Khamitova AD, Arapieva AM (2022) Imeglimin: features of the mechanism of action and potential benefits. Probl Endokrinol (Mosk) 68(3):57–66 35841169 10.14341/probl12868 PMC9762543

[CR72] Latimer CS, Burke BT, Liachko NF, Currey HN, Kilgore MD, Gibbons LE, Henriksen J, Darvas M, Domoto-Reilly K, Jayadev S, Grabowski TJ, Crane PK, Larson EB, Kraemer BC, Bird TD, Keene CD (2019) Resistance and resilience to Alzheimer’s disease pathology are associated with reduced cortical pTau and absence of limbic-predominant age-related TDP-43 encephalopathy in a community-based cohort. Acta Neuropathol Commun 7(1):91 31174609 10.1186/s40478-019-0743-1 PMC6556006

[CR73] Lazic SE (2015) Ranking, selecting, and prioritising genes with desirability functions. PeerJ 3:e1444 26644980 10.7717/peerj.1444 PMC4671156

[CR74] Li N, Zhou T, Fei E (2022) Actions of metformin in the brain: a new perspective of metformin treatments in related neurological disorders. Int J Mol Sci 23(15):8281 35955427 10.3390/ijms23158281 PMC9368983

[CR75] Lin MT, Beal MF (2006) Mitochondrial dysfunction and oxidative stress in neurodegenerative diseases. Nature 443(7113):787–795 17051205 10.1038/nature05292

[CR76] Lin H, Ao H, Guo G, Liu M (2023) The role and mechanism of metformin in inflammatory diseases. J Inflamm Res 16:5545 38026260 10.2147/JIR.S436147 PMC10680465

[CR77] Lista S, Imbimbo BP, Grasso M, Fidilio A, Emanuele E, Minoretti P, López-Ortiz S, Martín-Hernández J, Gabelle A, Caruso G, Malaguti M, Melchiorri D, Santos-Lozano A, Imbimbo C, Heneka MT, Caraci F (2024) Tracking neuroinflammatory biomarkers in Alzheimer’s disease: a strategy for individualized therapeutic approaches? J Neuroinflammation 21(1):187 39080712 10.1186/s12974-024-03163-y PMC11289964

[CR170] Lo D, Feng L, Li L, Carson MJ, Crowley M, Pauza M, Nguyen A, Reilly CR (1999) Integrating innate and adaptive immunity in the whole animal. Immunol Rev 169:225–239 10450520 10.1111/j.1600-065x.1999.tb01318.x

[CR78] Lu B, Nagappan G, Guan X, Nathan PJ, Wren P (2013) BDNF-based synaptic repair as a disease-modifying strategy for neurodegenerative diseases. Nat Rev Neurosci 14(6):401–416 23674053 10.1038/nrn3505

[CR79] Massa PT, Aleyasin H, Park DS, Mao X, Barger SW (2006) NFκB in neurons? The uncertainty principle in neurobiology. J Neurochem 97:607–618 16573643 10.1111/j.1471-4159.2006.03810.x PMC2063440

[CR80] Mattson MP, Magnus T (2006) Ageing and neuronal vulnerability. Nat Rev Neurosci 7(4):278–294 16552414 10.1038/nrn1886 PMC3710114

[CR81] Mattson MP, Goodman Y, Luo H, Fu W, Furukawa K (1997) Activation of NF-B protects hippocampal neurons against oxidative stress-induced apoptosis: evidence for induction of manganese superoxide dismutase and suppression of peroxynitrite production and protein tyrosine nitration. J Neurosci Res 49:681–697 9335256 10.1002/(SICI)1097-4547(19970915)49:6<681::AID-JNR3>3.0.CO;2-3

[CR83] Michailidis M, Tata DA, Moraitou D, Kavvadas D, Karachrysafi S, Papamitsou T, Vareltzis P, Papaliagkas V (2022) Antidiabetic drugs in the treatment of Alzheimer’s disease. Int J Mol Sci. 10.3390/ijms23094641 35563031 10.3390/ijms23094641 PMC9102472

[CR171] Mukhara D, Oh U, Neigh GN (2020) Chapter 15 – Neuroinflammation. In: Lanzenberger R, Kranz GS, Savic I (eds.) Handbook of Clinical Neurology, 175, Elsevier, pp 235-259 10.1016/B978-0-444-64123-6.00017-5 33008528

[CR84] Nagahara AH, Tuszynski MH (2011) Potential therapeutic uses of BDNF in neurological and psychiatric disorders. Nat Rev Drug Discov 10(3):209–219 21358740 10.1038/nrd3366

[CR85] Ní Chasaide C, Lynch MA (2020) The role of the immune system in driving neuroinflammation. Brain Neurosci Adv 4:239821281990108 10.1177/2398212819901082 PMC7085916 32219178

[CR86] Nieland TJ, Logan DJ, Saulnier J, Lam D, Johnson C, Root DE, Carpenter AE, Sabatini BL (2014) High content image analysis identifies novel regulators of synaptogenesis in a high-throughput RNAi screen of primary neurons. PLoS ONE 9(3):e91744 24633176 10.1371/journal.pone.0091744 PMC3954765

[CR87] Novack GD (2021) Repurposing medications. Ocul Surf 19:336–340 33249292 10.1016/j.jtos.2020.11.012 PMC7690306

[CR88] Ong LT, Sia CH (2025) Interactions between antidiabetes medications and heart–brain axis. Curr Opin Endocrinol Diabetes Obes 32(1):34–43 39639832 10.1097/MED.0000000000000896

[CR89] Onyango IG, Dennis J, Khan SM (2016) Mitochondrial dysfunction in Alzheimer’s disease and the rationale for bioenergetics-based therapies. Aging Dis 7(2):201–214 27114851 10.14336/AD.2015.1007 PMC4809610

[CR90] Ortega FJ, Gimeno-Bayon J, Espinosa-Parrilla JF, Carrasco JL, Batlle M, Pugliese M, Mahy N, Rodríguez MJ (2012) ATP-dependent potassium channel blockade strengthens microglial neuroprotection after hypoxia–ischemia in rats. Exp Neurol 235:282–296 22387180 10.1016/j.expneurol.2012.02.010

[CR91] Pai HC, Tzeng CY, Lee YC, Chang CH, Lin JG, Cheng JT, Chang SL (2009) Increase in plasma glucose lowering action of rosiglitazone by electroacupuncture at bilateral Zusanli acupoints (ST.36) in rats. J Acupunct Meridian Stud 2(2):147–151 20633486 10.1016/S2005-2901(09)60047-9

[CR92] Patel SS (2016) Cerebrovascular complications of diabetes: alpha glucosidase inhibitor as potential therapy. Horm Metab Res 48(2):83–91 26575305 10.1055/s-0035-1565181

[CR93] Paul S, Dansithong W, Gandelman M, Figueroa KP, Scoles DR, Pulst SM (2024) Cerebellar micro-RNA profile in a mouse model of spinocerebellar ataxia type 2. Neurol Genet 10:e200144 38715656 10.1212/NXG.0000000000200144 PMC11073881

[CR94] Pfützner A, Weber MM, Forst T (2007) Pioglitazone: update on an oral antidiabetic drug with antiatherosclerotic effects. Expert Opin Pharmacother 8(12):1985–1998 17696799 10.1517/14656566.8.12.1985

[CR95] Poor SR, Ettcheto M, Cano A, Sanchez-Lopez E, Manzine PR, Olloquequi J, Camins A, Javan M (2021) Metformin a potential pharmacological strategy in late onset Alzheimer’s disease treatment. Pharmaceuticals 14:890 34577590 10.3390/ph14090890 PMC8465337

[CR96] Qu Z, Titus ASCLS, Xuan Z, D’Mello SR (2018) Neuroprotection by heat shock factor-1 (HSF1) and trimerization-deficient mutant identifies novel alterations in gene expression. Sci Rep 8(1):17255 30467350 10.1038/s41598-018-35610-1 PMC6250741

[CR97] Rai SN, Singh C, Singh A, Singh MP, Singh BK (2020) Mitochondrial dysfunction: a potential therapeutic target to treat Alzheimer’s disease. Mol Neurobiol 57(7):3075–3088 32462551 10.1007/s12035-020-01945-y

[CR98] Ramakrishna K, Karuturi P, Siakabinga Q, TA G, Krishnamurthy S, Singh S, Kumari S, Kumar GS, Sobhia ME, Rai SN (2024) Indole-3 carbinol and diindolylmethane mitigated β-amyloid-induced neurotoxicity and acetylcholinesterase enzyme activity: in silico, in vitro, and network pharmacology study. Diseases 12(8):184 39195183 10.3390/diseases12080184 PMC11354007

[CR99] Ramos-Rodriguez JJ, Molina-Gil S, Ortiz-Barajas O, Jimenez-Palomares M, Perdomo G, Cozar-Castellano I, Lechuga-Sancho AM, Garcia-Alloza M (2014) Central proliferation and neurogenesis is impaired in type 2 diabetes and prediabetes animal models. PLoS ONE. 10.1371/journal.pone.0089229 24586614 10.1371/journal.pone.0089229 PMC3930705

[CR100] Ransohoff RM, Schafer D, Vincent A, Blachère NE, Bar-Or A (2015) Neuroinflammation: ways in which the immune system affects the brain. Neurotherapeutics 12:896–909 26306439 10.1007/s13311-015-0385-3 PMC4604183

[CR101] Rojo AI, Salinas M, Martín D, Perona R, Cuadrado A (2004) Regulation of Cu/Zn-superoxide dismutase expression via the phosphatidylinositol 3 kinase/Akt pathway and nuclear factor-κB. J Neurosci 24:7324–7334 15317858 10.1523/JNEUROSCI.2111-04.2004 PMC6729771

[CR102] Rosskothen-Kuhl N, Illing RB (2014) Gap43 transcription modulation in the adult brain depends on sensory activity and synaptic cooperation. PLoS ONE 9:e92624 24647228 10.1371/journal.pone.0092624 PMC3960265

[CR103] Rosskothen-Kuhl N, Illing RB (2015) The utilization of brain plasticity by cochlear implants: Molecular and cellular changes due to electrical intracochlear stimulation. HNO 63:94–103 (**German**) 25686598 10.1007/s00106-014-2976-4

[CR104] Roveta F, Bonino L, Piella EM, Rainero I, Rubino E (2024) Neuroinflammatory biomarkers in Alzheimer’s disease: from pathophysiology to clinical implications. Int J Mol Sci 25(22):11941 39596011 10.3390/ijms252211941 PMC11593837

[CR105] Roy Choudhury G, Ryou MG, Poteet E, Wen Y, He R, Sun F, Yuan F, Jin K, Yang SH (2014) Involvement of p38 MAPK in reactive astrogliosis induced by ischemic stroke. Brain Res 1551:45–58 24440774 10.1016/j.brainres.2014.01.013 PMC3987968

[CR106] Sandhu A, Rawat K, Gautam V, Kumar A, Sharma A, Bhatia A, Grover S, Saini L, Saha L (2024) Neuroprotective effect of PPAR gamma agonist in rat model of autism spectrum disorder: role of Wnt/β-catenin pathway. Prog Neuropsychopharmacol Biol Psychiatry 135:111126 39179196 10.1016/j.pnpbp.2024.111126

[CR172] Santiago JA, Potashkin JA (2023) Physical activity and lifestyle modifications in the treatment of neurodegenerative diseases. Front Aging Neurosci 15:1185671 37304072 10.3389/fnagi.2023.1185671 PMC10250655

[CR107] Schmid-Antomarchi H, Amoroso S, Fosset M, Lazdunski M (1990) K+ channel openers activate brain sulfonylurea-sensitive K+ channels and block neurosecretion. Proc Natl Acad Sci U S A 87:3489–3492 2333295 10.1073/pnas.87.9.3489 PMC53926

[CR108] Schmitt B, Bernhardt T, Moeller HJ, Heuser I, Frölich L (2004) Combination therapy in Alzheimer’s disease: a review of current evidence. CNS Drugs 18(13):827–844 15521788 10.2165/00023210-200418130-00001

[CR109] Selkoe DJ, Hardy J (2016) The amyloid hypothesis of Alzheimer’s disease at 25 years. EMBO Mol Med 8(6):595–608 27025652 10.15252/emmm.201606210 PMC4888851

[CR110] Shabanipour S, Jiao X, Rahimi-Balaei M, Aghanoori MR, Chung SH, Ghavami S, Consalez GG, Marzban H (2022) Upregulation of neural cell adhesion molecule 1 and excessive migration of Purkinje cells in cerebellar cortex. Front Neurosci 15:804402 35126044 10.3389/fnins.2021.804402 PMC8814629

[CR111] Sheng ZH (2017) The interplay of axonal energy homeostasis and mitochondrial trafficking and anchoring. Trends Cell Biol 27(6):403–416 28228333 10.1016/j.tcb.2017.01.005 PMC5440189

[CR112] Singh M, Agarwal V, Pancham P, Jindal D, Agarwal S, Rai SN, Singh SK, Gupta V (2024) A comprehensive review and androgen deprivation therapy and its impact on Alzheimer’s disease risk in older men with prostate cancer. Degener Neurol Neuromuscul Dis 14:33–46 38774717 10.2147/DNND.S445130 PMC11108066

[CR113] Singhal G, Jaehne EJ, Corrigan F, Toben C, Baune BT (2014) Inflammasomes in neuroinflammation and changes in brain function: a focused review. Front Neurosci 8:1–22 25339862 10.3389/fnins.2014.00315 PMC4188030

[CR173] Sivalingam S, Kandhasamy S, Chandrasekaran S, Vijayan K, Jacob JP, Perumal A, Vijayakumar S (2022) A flavone derivative from Andrographis echioides leaf extract positively alters the molecular targets of insulin signaling pathway. S Afr J Bot 146:760–770

[CR114] Sobral MVS, Soares VG, Moreira JLML, Rodrigues LK, Rocha P, Bendaham LCAR, Gonçalves OR, Pirolla RDC, Vilela LV, de Abreu VS, Almeida KJ (2025) The use of hypoglycemic drugs in Parkinson’s disease: an updated meta-analysis of randomized controlled trials. Parkinsonism Relat Disord 130:107210 39580237 10.1016/j.parkreldis.2024.107210

[CR115] Sola D, Rossi L, Schianca GPC, Maffioli P, Bigliocca M, Mella R, Corlianò F, Paolo Fra G, Bartoli E, Derosa G (2015) Sulfonylureas and their use in clinical practice. Arch Med Sci 11:840–848 26322096 10.5114/aoms.2015.53304 PMC4548036

[CR116] Song J, Yang Y, Mauvais-Jarvis F, Wang YP, Niu T (2017) KCNJ11, ABCC8 and TCF7L2 polymorphisms and the response to sulfonylurea treatment in patients with type 2 diabetes: a bioinformatics assessment. BMC Med Genet 18(1):64 28587604 10.1186/s12881-017-0422-7 PMC5461698

[CR117] Srivastava P, Tripathi PN, Sharma P, Rai SN, Singh SP, Srivastava RK, Shankar S, Shrivastava SK (2019) Design and development of some phenyl benzoxazole derivatives as a potent acetylcholinesterase inhibitor with antioxidant property to enhance learning and memory. Eur J Med Chem 163:116–135 30503937 10.1016/j.ejmech.2018.11.049

[CR118] Stafstrom CE, Rho JM (2012) The ketogenic diet as a treatment paradigm for diverse neurological disorders. Front Pharmacol 3:59 22509165 10.3389/fphar.2012.00059 PMC3321471

[CR119] Standards of Medical Care in Diabetes-2016: Summary of Revisions (2016). Diabetes Care 39(Suppl 1): S4–S5 10.2337/dc16-S003 26696680

[CR120] Stertz L, Magalhães PVS, Kapczinski F (2013) Is bipolar disorder an inflammatory condition? The relevance of microglial activation. Curr Opin Psychiatry 26:19–26 23196997 10.1097/YCO.0b013e32835aa4b4

[CR121] Su WJ, Peng W, Gong H, Liu YZ, Zhang Y, Lian YJ, Cao ZY, Wu R, Liu LL, Wang B, Wang YX, Jiang CL (2017) Antidiabetic drug glyburide modulates depressive-like behavior comorbid with insulin resistance. J Neuroinflammation. 10.1186/s12974-017-0985-4 29084550 10.1186/s12974-017-0985-4 PMC5663104

[CR122] Surmeier DJ, Obeso JA, Halliday GM (2017) Selective neuronal vulnerability in Parkinson disease. Nat Rev Neurosci 18(2):101–113 28104909 10.1038/nrn.2016.178 PMC5564322

[CR123] Swerdlow RH, Khan SM (2004) A “mitochondrial cascade hypothesis” for sporadic Alzheimer’s disease. Med Hypotheses 63(1):8–20 15193340 10.1016/j.mehy.2003.12.045

[CR124] Swerdlow RH, Burns JM, Khan SM (2014) The Alzheimer’s disease mitochondrial cascade hypothesis: progress and perspectives. Biochim Biophys Acta (BBA) - Mol Basis Dis 1842(8):1219–1231 10.1016/j.bbadis.2013.09.010 PMC3962811 24071439

[CR125] Tamura Y, Yamato M, Kataoka Y (2022) Animal models for neuroinflammation and potential treatment methods. Front Neurol 13:890217 35832182 10.3389/fneur.2022.890217 PMC9271866

[CR126] Tang F, Zhu D, Ma W, Yao Q, Li Q, Shi J (2021) Differences changes in cerebellar functional connectivity between mild cognitive impairment and Alzheimer’s disease: a seed-based approach. Front Neurol 12:645171 34220669 10.3389/fneur.2021.645171 PMC8248670

[CR127] Tang B, Wang Y, Jiang X, Thambisetty M, Ferrucci L, Johnell K, Hägg S (2022) Genetic variation in targets of antidiabetic drugs and Alzheimer disease risk: a mendelian randomization study. Neurology 99(7):e650 35654594 10.1212/WNL.0000000000200771 PMC9484609

[CR128] Tran J, Parekh S, Rockcole J, Wilson D, Parmar MS (2024) Repurposing antidiabetic drugs for Alzheimer’s disease: a review of preclinical and clinical evidence and overcoming challenges. Life Sci 355:123001 39173996 10.1016/j.lfs.2024.123001

[CR129] Tricco AC, Ashoor HM, Antony J, Bouck Z, Rodrigues M, Pham B, Khan PA, Nincic V, Darvesh N, Yazdi F, Ghassemi M, Ivory JD, Veroniki AA, Yu CH, Moja L, Straus SE (2021) Comparative efficacy and safety of ultra-long-acting, long-acting, intermediate-acting, and biosimilar insulins for type 1 diabetes mellitus: a systematic review and network meta-analysis. J Gen Intern Med 36:2414–2426 33742305 10.1007/s11606-021-06642-7 PMC8342652

[CR130] Tripathi PN, Srivastava P, Sharma P, Tripathi MK, Seth A, Tripathi A, Rai SN, Singh SP, Shrivastava SK (2019) Biphenyl-3-oxo-1,2,4-triazine linked piperazine derivatives as potential cholinesterase inhibitors with anti-oxidant property to improve the learning and memory. Bioorg Chem 85:82–96 30605887 10.1016/j.bioorg.2018.12.017

[CR131] Tripathi PN, Lodhi A, Rai SN, Nandi NK, Dumoga S, Yadav P, Tiwari AK, Singh SK, El-Shorbagi AA, Chaudhary S (2024) Review of pharmacotherapeutic targets in Alzheimer’s disease and its management using traditional medicinal plants. Degener Neurol Neuromuscul Dis 14:47–74 38784601 10.2147/DNND.S452009 PMC11114142

[CR174] Tupas GD, Otero MCB, Ebhohimen IE, Egbuna C, Aslam M (2020) Antidiabetic lead compounds and targets for drug development. In: Egbuna C, Kumar S, Ifemeje JC, Ezzat SM, Kaliyaperumal S (eds) Phytochemicals as lead compounds for new drug discovery, Elsevier, pp 127–141

[CR132] Vaughan EM, Santiago-Delgado ZM (2024) Management of type 2 diabetes mellitus with noninsulin pharmacotherapy. Am Fam Physician 109(4):333–342 38648832

[CR133] Verstraelen P, Van Dyck M, Verschuuren M, Kashikar ND, Nuydens R, Timmermans JP, De Vos WH (2018) Image-based profiling of synaptic connectivity in primary neuronal cell culture. Front Neurosci 12:389 29997468 10.3389/fnins.2018.00389 PMC6028601

[CR134] Vizuete AFK, Fróes F, Seady M, Zanotto C, Bobermin LD, Roginski AC, Wajner M, Quincozes-Santos A, Gonçalves CA (2022) Early effects of LPS-induced neuroinflammation on the rat hippocampal glycolytic pathway. J Neuroinflammation 19:1–23 36221097 10.1186/s12974-022-02612-w PMC9552490

[CR135] Wang YA, Kammenga JE, Harvey SC (2017) Genetic variation in neurodegenerative diseases and its accessibility in the model organism *Caenorhabditis elegans*. Hum Genomics 11(1):1–10 28545550 10.1186/s40246-017-0108-4 PMC5445269

[CR136] Wang Y, Hu H, Liu X, Guo X (2023) Hypoglycemic medicines in the treatment of Alzheimer’s disease: pathophysiological links between AD and glucose metabolism. Front Pharmacol 14:1138499 36909158 10.3389/fphar.2023.1138499 PMC9995522

[CR137] Wang N, Zhao Z, Wang X, Chen X, Jiang F, Tan Y, Chen W, Meng Q (2024) Brain regions differences in amyloid-β and gene expression in early APP/PS1 mice and identification of Npas4 as a key molecule in Alzheimer’s disease. Biomol Biomed 24(6):1816–1826 38958450 10.17305/bb.2024.10820 PMC11496853

[CR138] Wilcox CS (2020) Antihypertensive and renal mechanisms of SGLT2 (sodium-glucose linked transporter 2) inhibitors. Hypertension 75:894–901 32114848 10.1161/HYPERTENSIONAHA.119.11684

[CR175] Wrighten SA, Piroli GG, Grillo CA, Reagan LP (2009) A look inside the diabetic brain: contributors to diabetes-induced brain aging. Biochim Biophys Acta 1792:444–453 19022375 10.1016/j.bbadis.2008.10.013 PMC3991007

[CR139] Wu CY, Ouk M, Wong YY, Anita NZ, Edwards JD, Yang P, Shah BR, Herrmann N, Lanctôt KL, Kapral MK, MacIntosh BJ, Rabin JS, Black SE, Swardfager W (2020) Relationships between memory decline and the use of metformin or DPP4 inhibitors in people with type 2 diabetes with normal cognition or Alzheimer’s disease, and the role APOE carrier status. Alzheimers Dement 16(12):1663–1673 32803865 10.1002/alz.12161 PMC7754496

[CR140] Xu J, Patassini S, Rustogi N, Riba-Garcia I, Hale BD, Phillips AM, Waldvogel H, Haines R, Bradbury P, Stevens A, Faull RLM, Dowsey AW, Cooper GJS, Unwin RD (2019) Regional protein expression in human Alzheimer’s brain correlates with disease severity. Commun Biol 2:43 30729181 10.1038/s42003-018-0254-9 PMC6361956

[CR141] Yadav M, Dahiya M, Dagar J, Singh N, Sharma N, Rawat N, Dhakla P, Minocha N, Kumar A (2022) Role of mitochondria in brain functions and related disorders. Explor Med 3:494–515

[CR142] Yang SN, Berggren PO (2006) The role of voltage-gated calcium channels in pancreatic beta-cell physiology and pathophysiology. Endocr Rev 27:621–676 16868246 10.1210/er.2005-0888

[CR176] Yang T, Wang H, Li C, Duan H (2023) Mechanisms of drugs in the treatment of type 2 diabetes mellitus. Chin Med J (Engl) 136(4):394–396 36921103 10.1097/CM9.0000000000002356 PMC10106166

[CR143] Zhang W, Zhao L, Zhang J, Li P, Lv Z (2021) Metformin improves cognitive impairment in diabetic mice induced by a combination of streptozotocin and isoflurane anesthesia. Bioengineered 12:10982 34851228 10.1080/21655979.2021.2004978 PMC8809970

[CR144] Zhao JC, Zhang LX, Zhang Y, Shen YF (2012) The differential regulation of Gap43 gene in the neuronal differentiation of P19 cells. J Cell Physiol 227:2645–2653 21938722 10.1002/jcp.23006

[CR145] Zhu H, Zhang Y, Shi Z, Lu D, Li T, Ding Y, Ruan Y, Xu A (2016) The neuroprotection of Liraglutide against ischaemia-induced apoptosis through the activation of the PI3K/AKT and MAPK pathways. Sci Rep 6:26859 27240461 10.1038/srep26859 PMC4886514

[CR177] Zhu H, Jia Z, Li YR, Danelisen I (2023) Molecular mechanisms of action of metformin: latest advances and therapeutic implications. Clin Exp Med 23(7):2941–2951 37016064 10.1007/s10238-023-01051-y PMC10072049

[CR146] Zimmermann KS, Yamin JA, Rainnie DG, Ressler KJ, Gourley SL (2017) Connections of the mouse orbitofrontal cortex and regulation of goal-directed action selection by brain-derived neurotrophic factor. Biol Psychiatry 81:366–377 26786312 10.1016/j.biopsych.2015.10.026 PMC4871791

